# Subcellular localization of hippocampal ryanodine receptor 2 and its role in neuronal excitability and memory

**DOI:** 10.1038/s42003-022-03124-2

**Published:** 2022-03-01

**Authors:** Florian Hiess, Jinjing Yao, Zhenpeng Song, Bo Sun, Zizhen Zhang, Junting Huang, Lina Chen, Adam Institoris, John Paul Estillore, Ruiwu Wang, Henk E. D. J. ter Keurs, Peter K. Stys, Grant R. Gordon, Gerald W. Zamponi, Anutosh Ganguly, S. R. Wayne Chen

**Affiliations:** 1 Libin Cardiovascular Institute, Department of Physiology and Pharmacology, University of Calgary, Calgary, AB T2N 4N1 Canada; 2 Hotchkiss Brain Institute, Department of Physiology and Pharmacology, University of Calgary, Calgary, AB Canada; 3 Libin Cardiovascular Institute, Department of Cardiovascular Science, Department of Medicine, University of Calgary, Calgary, AB Canada; 4 Department of Clinical Neurosciences, University of Calgary, Calgary, AB Canada; 5 Department of Microbiology, Immunology, and Infectious Diseases, University of Calgary, Calgary, AB Canada

**Keywords:** Ion channels in the nervous system, Cellular neuroscience

## Abstract

Ryanodine receptor 2 (RyR2) is abundantly expressed in the heart and brain. Mutations in RyR2 are associated with both cardiac arrhythmias and intellectual disability. While the mechanisms of RyR2-linked arrhythmias are well characterized, little is known about the mechanism underlying RyR2-associated intellectual disability. Here, we employed a mouse model expressing a green fluorescent protein (GFP)-tagged RyR2 and a specific GFP probe to determine the subcellular localization of RyR2 in hippocampus. GFP-RyR2 was predominantly detected in the soma and dendrites, but not the dendritic spines of CA1 pyramidal neurons or dentate gyrus granular neurons. GFP-RyR2 was also detected within the mossy fibers in the stratum lucidum of CA3, but not in the presynaptic terminals of CA1 neurons. An arrhythmogenic RyR2-R4496C^+/−^ mutation downregulated the A-type K^+^ current and increased membrane excitability, but had little effect on the afterhyperpolarization current or presynaptic facilitation of CA1 neurons. The RyR2-R4496C^+/−^ mutation also impaired hippocampal long-term potentiation, learning, and memory. These data reveal the precise subcellular distribution of hippocampal RyR2 and its important role in neuronal excitability, learning, and memory.

## Abstract (web summary)

A mouse model containing a GFP-tagged ryanodine receptor 2 (RyR2) has shed light on the precise subcellular localization of hippocampal RyR2 and mechanisms underlying neuronal excitability, learning, and memory.

## Introduction

Ryanodine receptor type 2 (RyR2) is an intracellular Ca^2+^ channel that plays an essential role in excitation-contraction coupling in cardiac muscle by governing the release of Ca^2+^ from the sarcoplasmic reticulum^1^. RyR2 is also a critical player in the pathogenesis of cardiac arrhythmias. A large number of naturally occurring RyR2 mutations have been associated with catecholaminergic polymorphic ventricular tachycardia (CPVT), a malignant arrhythmia that can cause syncope or sudden death^2^. The arrhythmogenic mechanism of RyR2-associated CPVT has been extensively investigated. It is generally believed that CPVT-linked RyR2 mutations sensitize the channel to activation by Ca^2+^, which increases the propensity for spontaneous Ca^2+^ release, delayed afterdepolarization, and triggered arrhythmias^2^. In addition to the heart, RyR2 is the most abundantly expressed RyR isoform in the brain, especially in the hippocampus and cortex, regions that are important for learning/memory and cognition^3–6^. Interestingly, the level of RyR2 mRNA and protein in hippocampus was markedly increased following intensive training in a water maze task^7–10^. These observations suggest that RyR2 plays an important role in hippocampal synaptic plasticity and learning/memory. As such, altered RyR2 function is expected to have pathological impact on cognitive function. Indeed, apart from the arrhythmogenic phenotypes, a significant portion of patients harboring CPVT-linked RyR2 mutations also displayed intellectual disability (ID), cognitive deficits, and other neurodevelopmental disorders^11,12^. However, the mechanism by which CPVT RyR2 mutations affect cognitive function is poorly understood.

To gain insights into the roles of RyRs in neuronal function, numerous studies have investigated the cellular and subcellular localizations of RyRs in the brain. It has been shown that RyRs are expressed in different compartments in hippocampal neurons, including presynaptic terminals, dendritic spines, dendritic shafts, and the soma^6,13–17^. Consistent with these subcellular localizations of RyRs, functional studies have implicated an important role of RyRs in synaptic transmission, long-term potentiation (LTP), synaptic plasticity, and gene expression^14,18–26^. However, the precise subcellular localization and roles of the RyR2 isoform in different compartments (presynaptic terminals, dendritic spines, dendritic shafts, and the soma) in hippocampal neurons are largely undefined. This is due, in part, to the presence of all three RyR isoforms in hippocampal neurons, the uncertain antibody specificity in the context of whole brain tissue, and the lack of isoform-specific functional probes for RyR2.

To be able to specifically define the subcellular localization of the RyR2 isoform in the brain, here we used a knock-in (KI) mouse model that expresses a green fluorescent protein (GFP)-tagged RyR2. To increase the specificity and sensitivity of GFP detection, we generated a GFP-specific probe. Using these new reagents, we detected RyR2 in the soma and dendritic shafts, but not in the dendritic spines of hippocampal CA1 pyramidal neurons or dentate gyrus (DG) granular neurons. We also detected RyR2 within the mossy fibers in the stratum lucidum of CA3, but not in the presynaptic terminals of CA1 pyramidal neurons. Furthermore, to specifically assess the impact of CPVT RyR2 mutations, we employed a KI mouse model expressing a CPVT-linked human RyR2 mutation R4496C that enhances channel function^2,27–30^. We found that the RyR2-R4496C mutation decreased the A-type K^+^ current and increased the occurrence of spontaneous Ca^2+^ transients and action potential firing of CA1 pyramidal neurons, suggesting RyR2 as an important determinant of neuronal excitability. We also showed that the R4496C mutation impaired hippocampal LTP, learning and memory, but had no effect on afterhyperpolarization current or presynaptic short-term facilitation of CA1 pyramidal neurons. Our data reveal the precise subcellular localization of RyR2 in hippocampal CA1 pyramidal and DG granular neurons and the functional consequences of a CPVT RyR2 mutation in neuronal excitability and cognitive function.

## Results

### Localization of RyR2 in hippocampus using GFP-RyR2 knock-in mice and a novel GFP probe

**Fig. 1:**
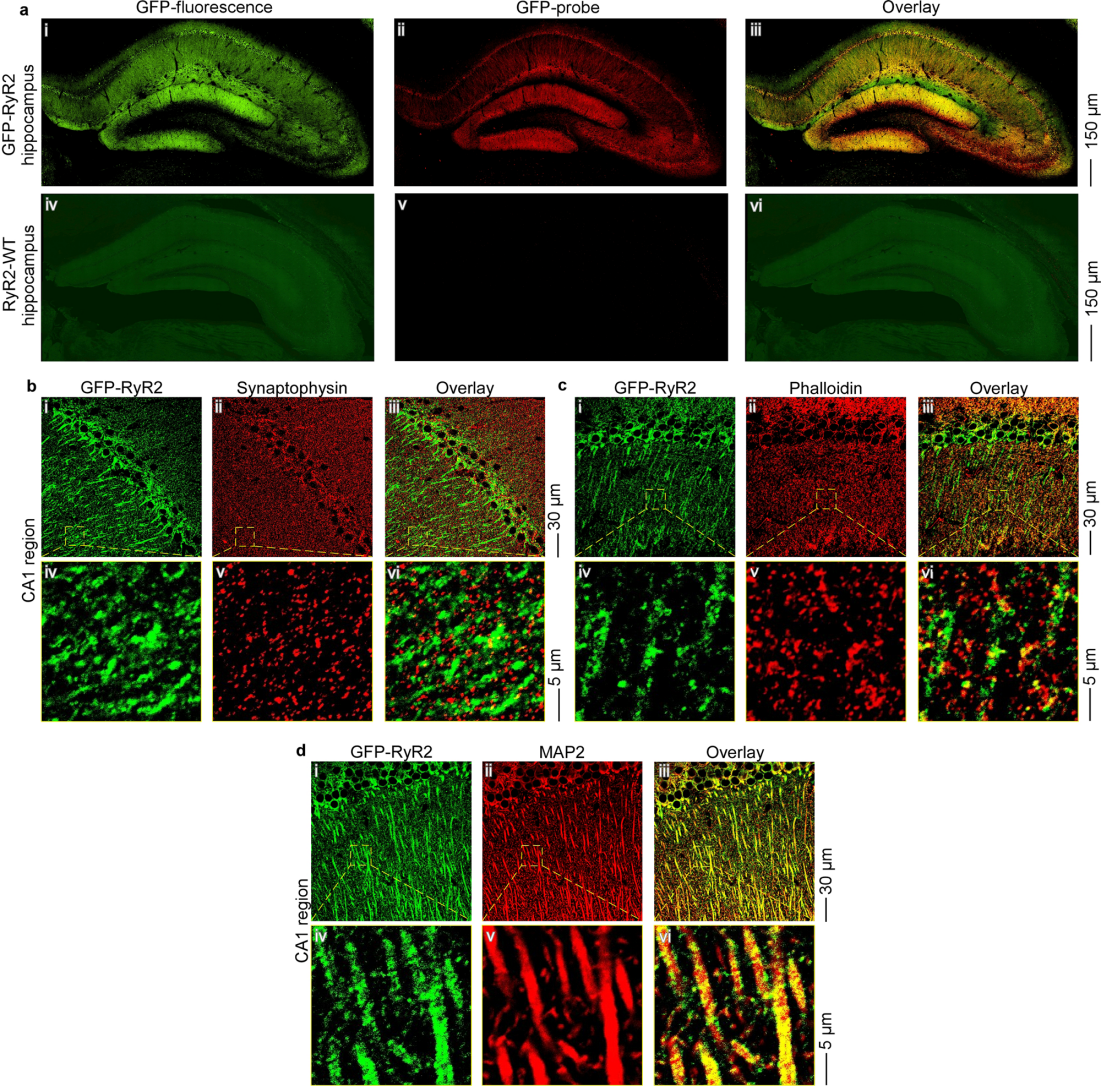
Co-localization of GFP-RyR2 with synaptic markers in hippocampal CA1 region in fixed brain slices using confocal imaging. **a** Representative confocal fluorescence images of hippocampus in fixed brain slices from mice expressing the GFP-tagged RyR2 (ai–iii) and non-GFP RyR2-WT mice (aiv–vi) (*n* = 16 images from eight brains for each group) with GFP-fluorescence (ai, iv), GFP-probe staining (aii, v), and overlay between the GFP and GFP-probe signals (aiii, vi) depicted. Representative confocal fluorescence images of hippocampal CA1 region in fixed brain slices from mice expressing a GFP-tagged RyR2 that were co-stained with the GFP-probe (displayed in green) and the presynaptic major vesicle protein synaptophysin (**b**), the F-actin targeting phalloidin (**c**), or the microtubule-associated protein 2 (MAP2) (**d**) (*n* = 30 images from eight brains). GFP-probe staining (i, iv), synaptic marker staining (ii, v), and overlay between the GFP-probe and synaptic marker signals (iii, vi) are depicted.

Given the presence of all three RyR isoforms in the brain, it is challenging to specifically localize RyR2. To circumvent this problem, we used a KI mouse model in which RyR2 is tagged by the GFP^31^. We performed confocal imaging of fixed brain slices from GFP-tagged RyR2 mice to directly visualize the location of GFP-RyR2 in hippocampus, a region known to be important for learning, memory, and cognition. As shown in Fig. 1, strong green fluorescence signals were detected in GFP-RyR2 hippocampus (Fig. 1ai), whereas only weak autofluorescence green signals were detected in the RyR2 wildtype (WT) (i.e., without GFP) hippocampus (Fig. 1aiv).

To minimize contribution of autofluorescence green signals and to increase the sensitivity and specificity of GFP-RyR2 detection, we generated a red fluorescent, GFP-specific probe in which a GFP-binding protein (GBP) (or GFP-nanobody)^32,33^ was fused to the C-terminus of the maltose-binding protein (MBP) (as the affinity tag). This MBP-GBP fusion protein was expressed in bacteria, purified, and labeled with Alexa Fluor 647 (AF647) dye. To test the specificity of this novel GFP probe, we incubated the GFP probe with isolated, fixed ventricular myocytes from the GFP-RyR2 mouse hearts. As expected, GFP-RyR2s in ventricular myocytes formed clusters with a highly ordered striated pattern (Supplementary Fig. 1a). Importantly, the signals of the red GFP probe detected in ventricular myocytes were superimposed with those of the GFP (Supplementary Fig. 1b, c). Similarly, the red GFP probe signals detected in the CA1 and DG regions were also superimposed with those of the GFP in the GFP-RyR2 brain slices (Fig. 1aii, iii and Supplementary Fig. 2a–c). On the other hand, little or no red GFP probe signals were detected in the same areas of WT (i.e., no GFP) (Fig. 1av, vi and Supplementary Fig. 1d–f). Taken together, these data indicate that the AF647-labeled MBP-GBP fusion protein is a highly specific probe for GFP-RyR2, and that RyR2 is abundantly expressed in hippocampal CA1 and DG neurons.

### Subcellular localization of RyR2 in hippocampal neurons using confocal imaging of fixed brain slices

To determine the subcellular distribution of GFP-RyR2 in hippocampal neurons, we performed co-localization analysis of GFP-RyR2 with well-established markers for different neuronal compartments in fixed hippocampal slices. There was little or no co-localization of GFP-RyR2 signals (as revealed by the GFP probe) with synaptophysin or syntaxin staining, both of which are well-established markers for presynaptic terminals, in hippocampal CA1 neurons (Fig. 1b and Supplementary Fig. 3a) and in granular cells of DG (Supplementary Figs. 3b and 4a). Similarly, there was little or no co-localization of the GFP probe with phalloidin staining, a well-established marker for dendritic spines^34–36^, in the hippocampal CA1 (Fig. 1c) and DG (Supplementary Fig. 4b) regions. In contrast, the GFP probe signals were extensively co-localized with the staining of the microtubule 2 associated protein 2 (MAP2), a well-established marker for postsynaptic structures (dendrites and soma), in the hippocampal CA1 (Fig. 1d) and DG (Supplementary Fig. 4c) regions. The GFP probe signals were clearly detected throughout dendritic shafts of CA1 pyramidal neurons and granular cells of DG (Fig. 1b–d and Supplementary Figs. 3 and 4). Thus, these co-staining studies suggest that RyR2 is mainly localized to the soma and dendritic shafts, but not the presynaptic terminals or the dendritic spines of hippocampal CA1 pyramidal or DG granular neurons.

### Subcellular localization of RyR2 in hippocampal neurons using structured illumination microscopy (SIM) imaging of fixed brain slices

To further define the subcellular distribution of RyR2, we carried out co-localization studies of GFP-RyR2 with well-established neuronal markers in fixed brain slices using structured illumination microscopy (SIM) imaging. As shown in Supplementary Figs. 5 and 6, SIM imaging revealed fine details of synaptophysin, phalloidin, MAP2, and GFP-probe staining in hippocampal CA1 (Supplementary Fig. 5) and DG (Supplementary Fig. 6) regions with staining patterns similar to those observed using confocal imaging. There was a minimal/partial overlap between the GFP probe signal and synaptophysin or phalloidin staining in hippocampal CA1 (Supplementary Fig. 5ai–iii, v–vii) or DG (Supplementary Fig. 6ai–iii, v–vii) regions. In contrast, there was an extensive overlap of the GFP probe signal and MAP2 staining in hippocampal CA1 (Supplementary Fig. 5aix–xi) and DG (Supplementary Fig. 6aix–xi) regions. Quantitative analysis of color overlap in SIM images showed that there was ~50% overlap between the GFP probe and MAP2 signals, whereas, there was <5% overlap between the GFP probe and synaptophysin or phalloidin signals in CA1 and DG regions (Supplementary Figs. 5aiv, viii, xii, 5b and 6aiv, viii, xii, 6b). Thus, in agreement with confocal imaging analysis, SIM imaging analysis also suggests the localization of RyR2 mainly in the soma and dendrites, but not the presynaptic terminals or dendritic spines, of hippocampal CA1 pyramidal or DG granular neurons.

### Subcellular localization of RyR2 in tdTomato-filled hippocampal neurons using confocal imaging of live brain slices

**Fig. 2:**
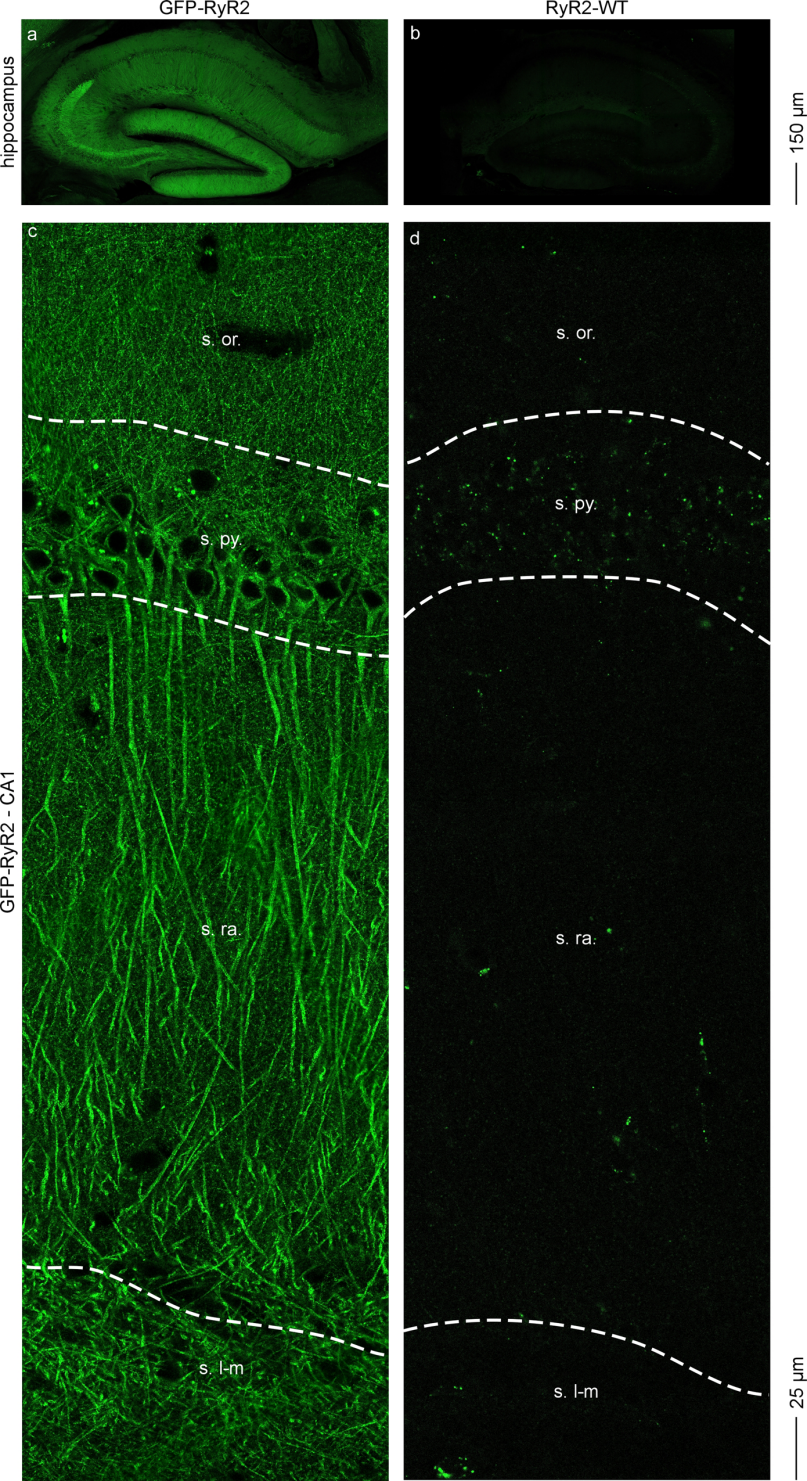
Confocal imaging of live hippocampal slices from GFP-RyR2 and non-GFP RyR2 WT mice. Representative confocal GFP fluorescence images of live hippocampal slices from mice expressing the GFP-tagged RyR2 (**a**, **c**) and non-GFP RyR2 WT mice (**b**, **d**). Images of the whole hippocampal area of the GFP-RyR2 (**a**) and WT (**b**) brains. Magnified view showing the stratum oriens (s. or.), stratum pyramidate (s. py.), stratum radiatum (s. ra.), and stratum lacunosum-moleculare (s. l-m) areas of the CA1 region in GFP-RyR2 (**c**) and WT (**d**) live hippocampal slices (*n* = 9 images from three brains for each group).

Although fixed brain slices are necessary for studying co-localization of GFP-RyR2 with neuronal markers using immunostaining techniques, fixation itself could potentially affect neuronal structures and the GFP fluorescence. To avoid potential fixation-induced perturbations, we performed confocal imaging of acute, live hippocampal slices (without fixation). As shown in Fig. 2, strong green fluorescence signals were detected in the GFP-RyR2 hippocampus (Fig. 2a), but were hardly detected in the non-GFP RyR2-WT hippocampus (Fig. 2b). With a higher magnification, green signals were readily detected in all areas of the GFP-RyR2 hippocampal CA1 region, including the stratum oriens (s. or.), stratum pyramidale (s. py.), stratum radiatum (s. ra.), and stratum lacunosum-moleculare (s. l-m), especially in the soma and dendrites (Fig. 2c). In sharp contrast, little or no green signal was detected in any area of the non-GFP RyR2 WT hippocampal CA1 region (Fig. 2d). There were some bright green dots present in the s. py. area of both the GFP-RyR2 and non-GFP RyR2 hippocampi (Fig. 2c, d).

**Fig. 3:**
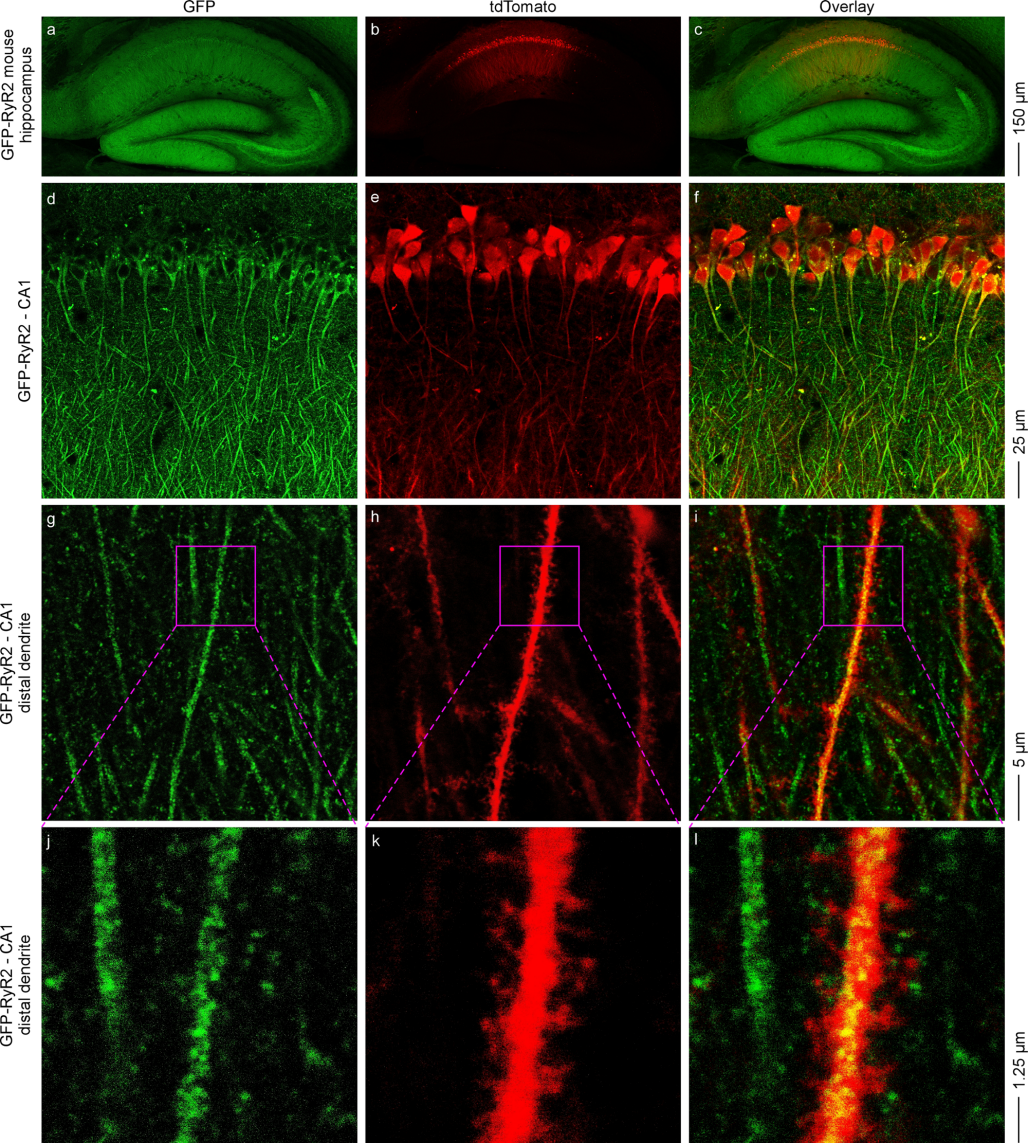
Confocal imaging of live hippocampal slices from GFP-RyR2 mice with AAV9-tdTomato infected CA1 region. Representative confocal fluorescence images of live hippocampal slices from mice expressing the GFP-tagged RyR2 with AAV9-tdTomato infected CA1 region (*n* = 9 images from three brains). Images of the whole hippocampal area showing the AAV9-tdTomato infected CA1 region (**a**–**c**). Magnified views showing the CA1 area (**d**–**f**), a single tdTomato-filled distal dendrite (**g**–**i**), and the dendritic spines of the tdTomato-filled distal dendrite (**j**–**l**).

We next employed this in situ confocal imaging of live brain slices to directly visualize the distribution of GFP-RyR2 in different subcellular compartments. To this end, we infected hippocampal CA1 neurons with the hSYN1 promoter-driven tdTomato-expressing AAV9 virus to fill the cells (Fig. 3a–c). To facilitate the identification of subcellular compartments, a low titer of the AAV9-tdTomato virus was injected into the GFP-RyR2 hippocampal CA1 region to infect only a few well separated individual neurons. We then performed confocal dual (GFP and tdTomato) imaging of acute, live hippocampal slices. As shown in Fig. 3, multiple tdTomato-filled cells were detected in the CA1 region (Fig. 3d–f). A closer examination of CA1 distal dendrites with higher magnification revealed numerous dendritic spines. An example of a tdTomato-filled dendritic shaft and spines is shown in Fig. 3h, k. Notably, strong GFP signals were detected in the soma and dendritic shafts (Fig. 3d, g, j). Importantly, little or no GFP signals were detected in any well resolved tdTomato-filled dendritic spine of any tdTomato-filled dendrite examined (*n* = 9 images from three brains). An example of lack of detectable GFP signals in tdTomato-filled dendritic spines is shown in Fig. 3j–l. Note that no GFP signals were detected in the AAV9-tdTomato injected CA1 region of live non-GFP RyR2 brain slices (Supplementary Fig. 7), indicating the specificity of the GFP signals. Also note that GFP-RyR2 clusters were detected in both the dendritic shafts and dendritic branch points (Supplementary Fig. 8). However, the limited resolution of our confocal GFP imaging of live brain slices did not permit quantitative analysis of the expression and distribution of GFP-RyR2 clusters at different dendritic locations to assess whether RyR2 clusters are preferentially localized to the dendritic branch points^37,38^.

We also performed two-photon dual imaging of tdTomato-filled, GFP-RyR2 CA1 pyramidal neurons in acute, live hippocampal slices and obtained similar results (Supplementary Fig. 9). Therefore, consistent with our co-localization analysis, these cell-filling, dual imaging studies revealed that GFP-RyR2 was mainly localized to the soma and dendritic shafts, but not the dendritic spines of hippocampal CA1 pyramidal neurons.

**Fig. 4:**
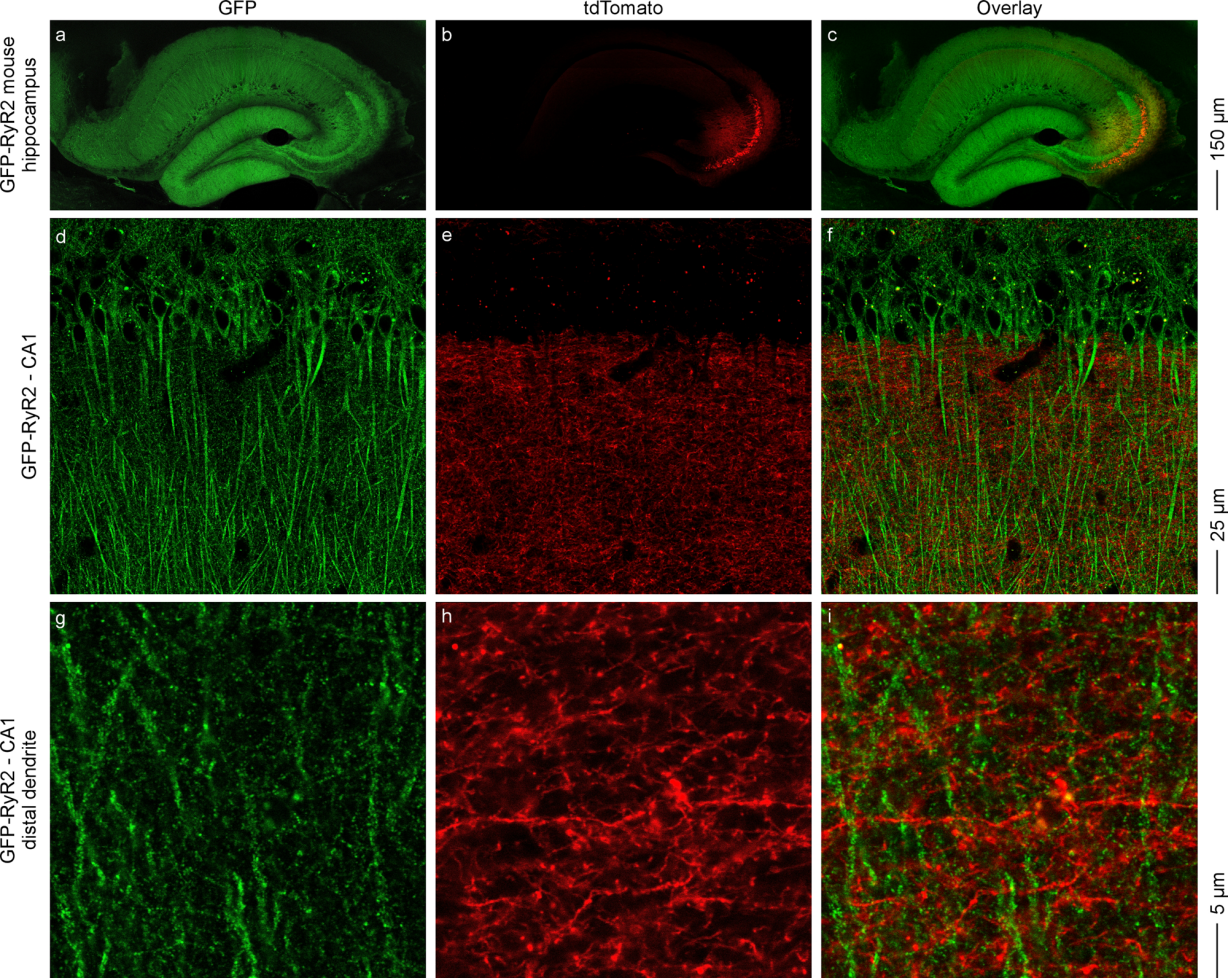
Confocal imaging of live hippocampal slices from GFP-RyR2 mice with AAV9-tdTomato infected CA3 region. Representative confocal fluorescence images of live hippocampal slices from mice expressing the GFP-tagged RyR2 with AAV9-tdTomato infected CA3 region (*n* = 9 images from three brains). Images of the whole hippocampal area showing the AAV9-tdTomato infected CA3 region (**a**–**c**). Magnified views showing the CA1 area (**d**–**f**) and tdTomato-filled axons from the CA3 region and distal dendrites of CA1 neurons (**g**–**i**).

CA3 pyramidal neurons are known to project their axons to the CA1 pyramidal neurons^39–41^. To determine whether RyR2 is expressed in the presynaptic terminals of CA1 pyramidal neurons, we injected AAV9-tdTomato viruses into the GFP-RyR2 mouse hippocampal CA3 region in order to visualize the axonal projections from CA3 pyramidal neurons to the distal stratum radiatum (s. ra) area of the CA1 region. As shown in Fig. 4, a number of tdTomato-filled cells were detected in the CA3 region (Fig. 4a–c). We also detected a dense mesh of tdTomato-filled thin axonal processes in the CA1 region (Fig. 4e, f) and strong GFP signals in the soma and dendritic shafts of the GFP-RyR2 CA1 pyramidal neurons (Fig. 4d, g). A closer examination of the CA1 distal stratum radiatum area with higher magnification revealed little overlap of GFP and tdTomato signals in any image examined (*n* = 9 images from three brains). An example of lack of overlap of GFP and tdTomato signals is shown in Fig. 4g–i. No GFP signals were detected in the CA1 region of live non-GFP RyR2 brain slices after AAV9-tdTomato injection into the CA3 region (Supplementary Fig. 10). Thus, consistent with our co-localization analysis, cell-filling and dual imaging studies revealed that GFP-RyR2 was mainly localized to the soma and dendritic shafts, but not the presynaptic terminals of hippocampal CA1 pyramidal neurons.

We employed the same approach to visualizing the subcellular distribution of GFP-RyR2 in granular neurons of the DG. We injected tdTomato-expressing AAV9 viruses into the GFP-RyR2 mouse DG region (Supplementary Fig. 11a–c). A number of tdTomato-filled cells were detected in the DG region (Supplementary Fig. 11d–f). A closer examination of DG distal dendrites with higher magnification revealed numerous dendritic spines. An example of a tdTomato-filled DG dendritic shaft and spines is shown in Supplementary Fig. 11h, k. As with CA1 pyramidal neurons, strong GFP signals were detected in the soma and dendritic shafts (Supplementary Fig. 11d, g, j). Similarly, little or no GFP signals were detected in any well resolved tdTomato-filled dendritic spine of any tdTomato-filled dendrite examined (*n* = 9 images from three brains). An example of lack of detectable GFP signals in tdTomato-filled dendritic spines is shown in Supplementary Fig. 11j–l. Note that there were no GFP signals detected in the DG region of live non-GFP RyR2 brain slices injected with AAV9-tdTomato viruses (Supplementary Fig. 12). Taken together, these cell-filling and dual imaging studies revealed that, as with CA1 pyramidal neurons, GFP-RyR2 was also predominantly localized to the soma and dendritic shafts, but not the dendritic spines of DG granular neurons.

We also examined the distribution of GFP-RyR2 in the axonal projections from DG granular neurons after injecting tdTomato-expressing AAV9 viruses into the GFP-RyR2 mouse DG region (Supplementary Fig. 13a–c). A closer examination of the tdTomato-filled large mossy fibers projecting from the DG region to the stratum lucidum area of the CA3 region with higher magnification revealed many small RyR2 clusters along the mossy fibers (Supplementary Fig. 13d–i), which gave rise to an intense GFP-RyR2 staining in the stratum lucidum of CA3 (Supplementary Fig. 13a–c). This suggests that GFP-RyR2 is also expressed in the CA3 mossy fibers, which is consistent with that reported previously^16^.

### The R4496C^+/−^ mutation increases spontaneous Ca^2+^ transients in hippocampal CA1 neurons

**Fig. 5:**
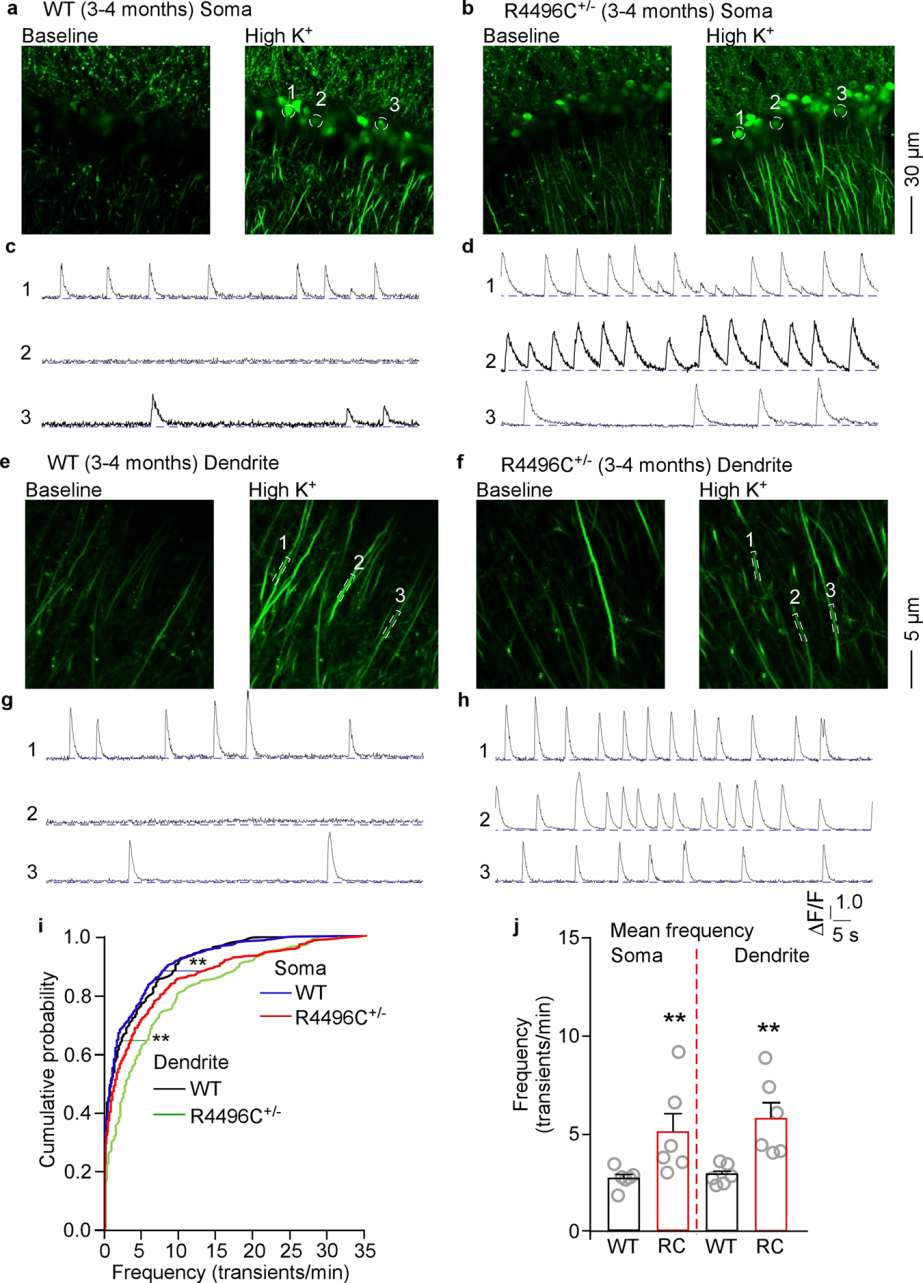
CPVT RyR2 mutation R4496C^+/−^ enhances spontaneous Ca^2+^ transients in hippocampal CA1 neurons. Hippocampal brain slices were prepared from RyR2 WT/GCaMP6f^+/−^ and RyR2-R4496C^+/−^/GCaMP6f^+/−^ mice (3–4 months old) expressing the GCaMP6f Ca^2+^ sensing probe. GCaMP6f fluorescence signals from individual hippocampal CA1 neurons were continuously recorded at the resting condition (spontaneous Ca^2+^ transients) for 5 min, followed by the addition of KCl (50 mM) (KCl-induced Ca^2+^ transients) using the Leica SP8 DIVE up-right two-photon imaging system. Note that here KCl depolarization was applied to the brain slices after finishing recording of spontaneous Ca^2+^ transients at rest to identify viable neurons. GCaMP6f fluorescence images of hippocampal CA1 neurons in RyR2 WT/GCaMP6f^+/−^ (**a**) and RyR2-R4496C^+/−^/GCaMP6f^+/−^ (**b**) mouse brain slices at rest (baseline) and after KCl perfusion. **c**, **d** GCaMP6f fluorescence traces of the three neurons circled in **a** and **b** at rest/baseline (i.e., without KCl perfusion), respectively. GCaMP fluorescence images of hippocampal CA1 neuronal distal dendrites in RyR2 WT/GCaMP6f^+/−^ (**e**) and RyR2-R4496C^+/−^/GCaMP6f^+/−^ (**f**) mouse brain slices at rest and after KCl perfusion. **g**, **h** GCaMP6f fluorescence traces of the three dendrites circled in **e**, **f**, respectively, at rest/baseline (i.e., without KCl perfusion). **i** Cumulative probability functions showing frequency distributions of spontaneous Ca^2+^ transients at rest in the soma (blue) and dendrite (black) of CA1 neurons of 3–4 months old RyR2 WT/GCaMP6f^+/−^ mice and in the soma (red) and dendrite (green) of CA1 neurons of 3–4 months old RyR2-R4496C^+/−^/GCaMP6f^+/−^ mice (Kruskal–Wallis test with Dunn’s multiple comparisons test). **j** Mean frequency of spontaneous Ca^2+^ transients at rest in the soma and dendrite area of CA1 neurons of 3–4 months old RyR2 WT/GCaMP6f^+/−^ and RyR2-R4496C^+/−^/GCaMP6f^+/−^ mice. Data shown are mean ± SEM (*n* = 18 slices from 6 RyR2 WT/GCaMP6f^+/−^ brains with 600 somas, 182 dendrites, and *n* = 18 slices from 6 RyR2-R4496C^+/−^ brains with 469 somas and 159 dendrites) (Mann–Whitney *U* test, **p < 0.01).

Arrhythmogenic RyR2 mutations have been associated with impaired cognitive function, but the underlying mechanism is unclear. To this end, we determined the impact of a CPVT-causing RyR2 mutation R4496C^+/−^, which enhances the sensitivity of RyR2 to Ca^2+^ activation^2,27–30^, on neuronal excitability, a critical determinant of learning and memory^42–44^. We crossed heterozygous RyR2 R4496C^+/−^ mutant mice with heterozygous Thy1-GCaMP6f^+/−^ transgenic mice^45^ to produce RyR2 WT/GCaMP6f^+/−^ and RyR2-R4496C^+/−^/GCaMP6f^+/−^ mice. Under the control of the Thy1 promoter, the GCaMP6f protein, a genetically encoded, fast, ultrasensitive fluorescence Ca^2+^ indicator, is expressed in hippocampal and cortical neurons. We performed two-photon in vitro imaging of GCaMP6f-expressing CA1 neurons in acute brain slices to monitor spontaneous Ca^2+^ transients, which have been widely used to assess the spontaneous neuronal activity of cell populations^45–49^. As shown in Fig. 5, among the hippocampal CA1 neurons that responded to KCl depolarization, the frequency of these spontaneous Ca^2+^ transients in both the soma (Fig. 5a–d, i, j) and dendritic shafts (Fig. 5e–j) in RyR2 R4496C^+/−^ mutant brain slices at rest (i.e., without KCl perfusion) was markedly increased compared to those in WT. Note that here KCl depolarization was applied to the brain slices after finishing recording of spontaneous Ca^2+^ transients at rest to identify viable neurons. Thus, the arrhythmogenic RyR2 R4496C^+/−^ mutation increases the occurrence of spontaneous Ca^2+^ transients, which is consistent with increased neuronal excitability.

### The R4496C^+/−^ mutation increases spontaneous and triggered action potential (AP) firing in hippocampal CA1 neurons

**Fig. 6:**
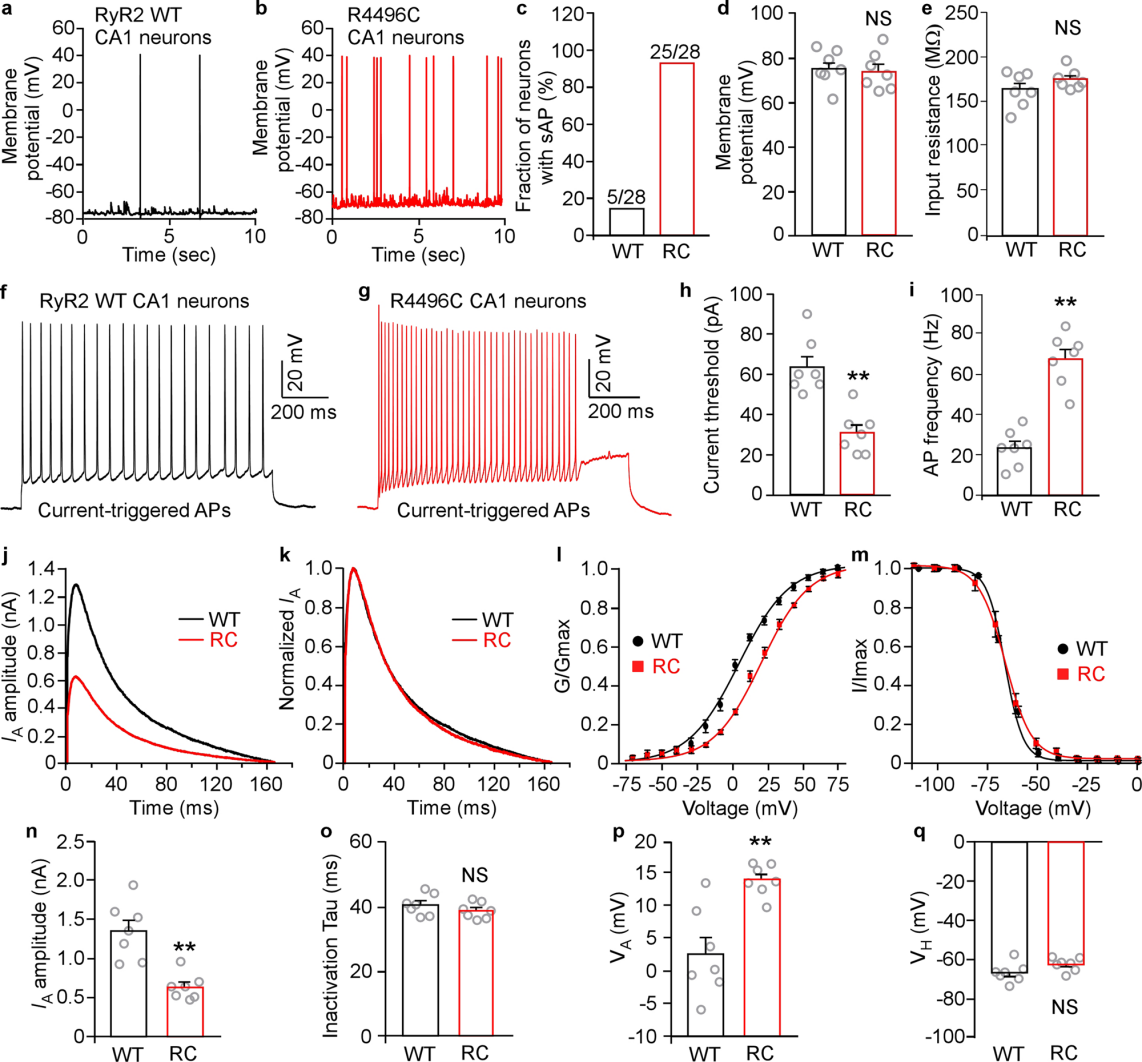
CPVT RyR2 mutation R4496C^+/−^ enhances spontaneous and triggered action potential firing and decreases the A-type K^+^ current (*I*_A_) in hippocampal CA1 neurons. Hippocampal brain slices were prepared from RyR2 WT and R4496C^+/−^ mutant mice (3–4 months old). Action potential firing was recorded using whole-cell patch-clamp technique. Representative traces of membrane potential recordings showing spontaneous action potential (sAP) firing in RyR2 WT (**a**) and R4496C^+/−^ mutant (**b**) mouse hippocampal CA1 neurons. **c** Fraction (%) of RyR2 WT and R4496C^+/−^ (RC) CA1 neurons displaying sAP firing. Note that the RyR2 mutation R4496C^+/−^ markedly increases the percentage of CA1 neurons showing sAP firing. **d** Resting membrane potentials of CA1 neurons from RyR2 WT and R4496C^+/−^ mutant mice (3–4 months old). **e** Input resistance of CA1 neurons from RyR2 WT and R4496C^+/−^ mutant mice (3–4 months old). Representative traces of membrane potential recordings showing AP firing after current injection of 150 pA into the RyR2 WT (**f**) and R4496C^+/−^ mutant (**g**) hippocampal CA1 neurons. **h** Current threshold (pA) was defined as the minimum current injection required to trigger the first AP firing in WT or R4496C^+/−^ (RC) mouse hippocampal CA1 neurons. Note that the R4496C^+/−^ mutation reduces the current threshold for triggering AP firing. **i** The frequency of current injection-triggered APs. Note that the R4496C^+/−^ mutation increases the AP firing frequency in hippocampal CA1 neurons. Data shown are mean ± SEM (*n* = 28 neurons in slices from 7 WT brains, and *n* = 28 neurons in slices from 7 R4496C^+/−^ brains) (Mann–Whitney *U* test, *p < 0.05). **j** Representative traces of A-type K^+^ current (*I*_A_) from 3–4 months old RyR2 WT and R4496C^+/−^ (RC) CA1 neurons. **k** Normalized *I*_A_ traces. **l** Steady-state activation curves of *I*_A_. **m** Steady-state inactivation curves of *I*_A_. **n** *I*_A_ amplitude in 3–4 months old RyR2 WT (7 mice, 28 neurons) and R4496C^+/−^ (RC) (7 mice, 28 neurons) CA1 neurons. **o** *I*_A_ inactivation kinetics (Tau) from the same number of neurons as in **n**. **p** The midpoints of voltage-dependent activation of *I*_A_ (*V*_A_) for RyR2 WT (7 mice, 28 neurons) and R4496C^+/−^ (RC) (7 mice, 28 neurons) CA1 neurons. **q** The midpoints of voltage-dependent inactivation of *I*_A_ (*V*_H_) for RyR2 WT (7 mice, 28 neurons) and R4496C^+/−^ (RC) (7 mice, 28 neurons) CA1 neurons. (Mann–Whitney *U* test, **p < 0.05; NS not significant).

To directly assess the impact of the arrhythmogenic RyR2 R4496C^+/−^ mutation on neuronal excitability, we performed whole-cell patch-clamp recordings of hippocampal CA1 pyramidal neurons in brain slices from the RyR2 WT and R4496C^+/−^ mutant mice. As shown in Fig. 6, spontaneous AP firings were detected in both the WT and R4496C^+/−^ mutant hippocampal CA1 pyramidal neurons. However, the fraction of CA1 pyramidal neurons that displayed spontaneous AP firing was substantially higher in R4496C^+/−^ brain slices than in WT (Fig. 6a–c) (*p* < 0.05). There was no significant difference in the resting membrane potential or input resistance between RyR2 WT and R4496C^+/−^ mutant CA1 pyramidal neurons (Fig. 6d, e). Furthermore, the threshold of current injection required to trigger AP firing in the R4496C^+/−^ mutant CA1 pyramidal neurons was significantly lower than that needed to trigger AP firing in WT neurons (Fig. 6f–h) (*p* < 0.05). The frequency of current injection-triggered APs in R4496C^+/−^ mutant CA1 pyramidal neurons was also markedly increased compared to that in WT (Fig. 6f, g, i) (*p* < 0.05). These data demonstrate that the arrhythmogenic R4496C^+/−^ mutation increases neuronal excitability of hippocampal CA1 neurons.

### The R4496C^+/−^ mutation decreases the A-type K^+^ current of hippocampal CA1 neurons, but has no effect on afterhyperpolarization current

The A-type K^+^ current is a well-known control of excitability of CA1 pyramidal neurons^50–54^. To further investigate the mechanism by which the R4496C^+/−^ mutation increases neuronal excitability, we determined the impact of the RyR2 R4496C^+/−^ mutation on the A-type K^+^ current. We assessed the A-type K^+^ current in hippocampal CA1 neurons in brain slices from RyR2 WT and the R4496C^+/−^ mutant mice using whole-cell patch-clamp recordings. As shown in Fig. 6, the R4496C^+/−^ mutation markedly downregulated the A-type K^+^ current of hippocampal CA1 neurons (Fig. 6j–q). Specifically, the R4496C^+/−^ mutation decreased the amplitude (Fig. 6j, n) of the A-type K^+^ current and shifted the half-activation voltage (VA) rightward (Fig. 6l, p) without altering the decay time (inactivation Tau) or half-inactivation voltage (VH) (Fig. 6k, m, o, q). Thus, the arrhythmogenic R4496C^+/−^ mutation decreases the A-type K^+^ current thereby increasing neuronal excitability.

We also measured the afterhyperpolarization current (*I*_AHP_) in CA1 pyramidal neurons because *I*_AHP_ influences intrinsic excitability^55–57^. There was no significant difference in either the medium *I*_AHP_ (*I*_mAHP_) or slow *I*_AHP_ (*I*_sAHP_) between RyR2-WT and RyR2-R4496C^+/−^ mutant CA1 neurons (Supplementary Fig. 14). Thus, the action of the R4496C^+/−^ mutation in intrinsic excitability is unlikely to be mediated by *I*_AHP_.

### The R4496C^+/−^ mutation impairs hippocampal long-term potentiation (LTP)

**Fig. 7:**
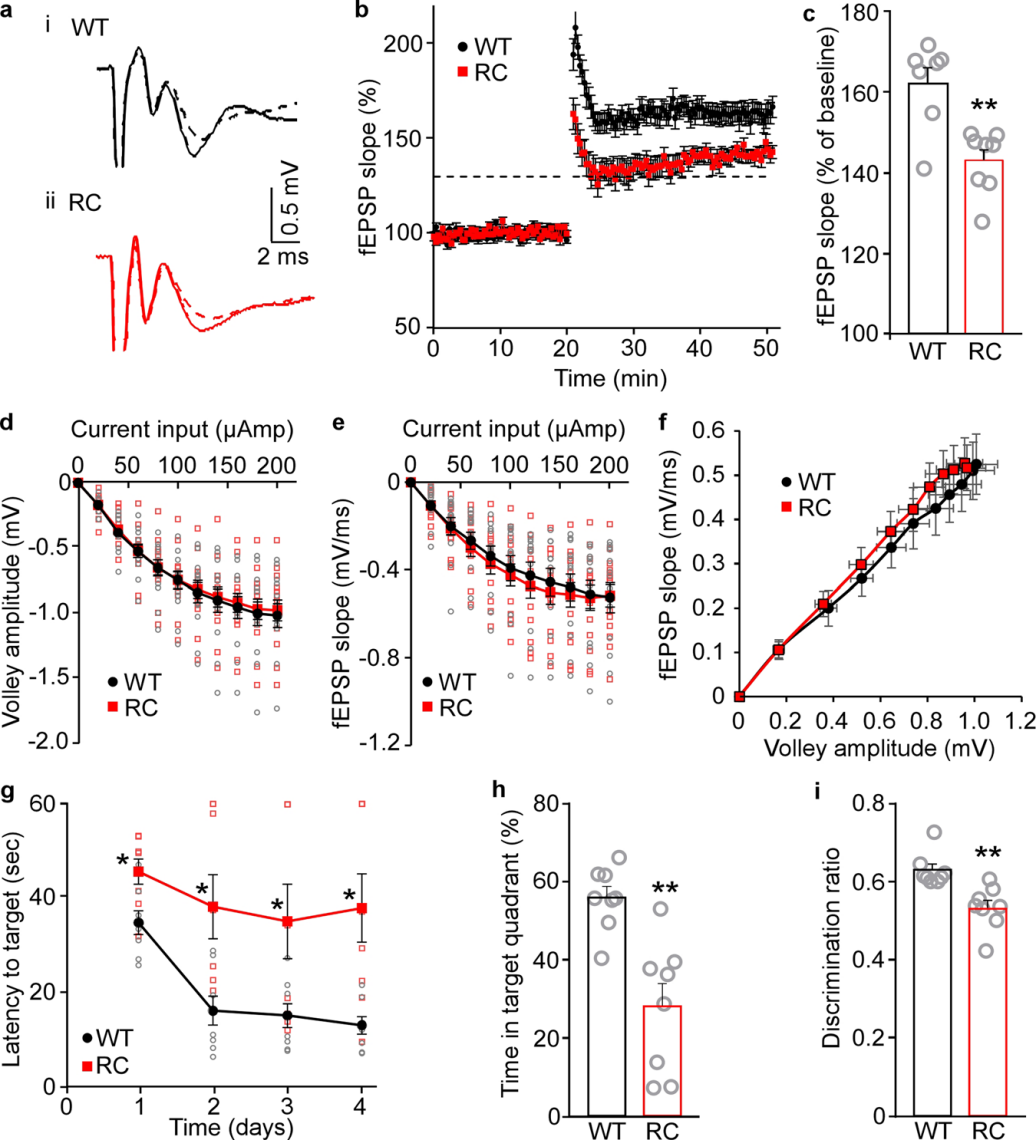
Effects of the arrhythmogenic RyR2 R4496C^+/−^ mutation on long-term potentiation (LTP) and spatial learning and memory. **a** Representative fEPSP traces under basal conditions (dashed lines) and after HFS (solid lines) in RyR2-WT (**a**i) and R4496C^+/−^ mutant (**a**ii) brain slices (3–4 months old). **b** fEPSP slopes in Schaffer collaterals in both the RyR2-WT and R4496C^+/−^ mutant brain slices remained increased (≥130% indicated by the dashed line) for at least 30 min after HFS. **c** Average fEPSP slopes in RyR2-WT (WT) and R4496C^+/−^ (RC) mutant brain slices (at 40–50 min). Note that there are no significant differences in the amplitude of fiber volleys (**d**) and fEPSP slopes (**e**) in relation to the current input or fiber volley amplitude against the fEPSP slope (**f**) between RyR2-WT and R4496C^+/−^ mutant brain slices. Data shown are mean ± SEM (*n* = 14 brain slices from 7 WT, and 15 brain slices from 8 R4496C^+/−^) (Mann–Whitney *U* test, *p < 0.01). Spatial learning and memory were assessed using the Morris water maze (MWM) and the Novel Object Preference (NOP) tests. **g** The latency to reach the target escape platform during the training period (days 1–4) in the MWM test. **h** Time spent in the target quadrant after removing the platform during the probing period 24-h after the last training session in the MWM test. **i** The percentage of time spent in the novel object during the NOP test. Data shown are mean ± SEM (*n* = 8 for WT and 8 for R4496C^+/−^) (Mann–Whitney *U* test, *p < 0.05).

We next determined whether the arrhythmogenic RyR2 R4496C^+/−^ mutation affects hippocampal LTP, a well-known mechanism involved in learning and memory^58,59^. To this end, we measured LTP at Schaffer collateral – CA1 synapses in brain slices from the RyR2 WT and R4496C^+/−^ mutant mice. As shown in Fig. 7, fEPSP slopes of Schaffer collaterals in both the RyR2 WT and R4496C^+/−^ mutant brain slices remained increased (≥130%) for at least 30 min after a train of high frequency stimulation (HFS). This indicates the presence of LTP (Fig. 7a, b). However, the extent of LTP was significantly reduced in the R4496C^+/−^ mutant compared to that in WT (Fig. 7c). There were no significant differences in the amplitudes of fiber volleys or fEPSP slopes in relation to the current input between WT and R4496C^+/−^ mutant brain slices, also no significant difference in the fiber volley amplitude against the fEPSP slope (Fig. 7d–f). Thus, the arrhythmogenic RyR2 R4496C^+/−^ mutation impairs hippocampal LTP.

### The R4496C^+/−^ mutation impairs learning and memory

Given the close link between hippocampal LTP and learning/memory, the RyR2 R4496C^+/−^ mutation that impairs hippocampal LTP may also affect learning and memory. To test this, we performed Morris water maze (MWM) tests on 3–4 months old RyR2 WT and the R4496C^+/−^ mutant mice to evaluate their spatial learning and memory. As shown in Fig. 7, the RyR2 R4496C^+/−^ mice required significantly longer time than WT to find the submerged platform (Fig. 7g) and spent significantly less time in the target quadrant in the probe test 24 h after the last training session (Fig. 7h). It should be noted that there was no difference in the mean swim speed between RyR2 WT and the R4496C^+/−^ mutant mice (Supplementary Fig. 15a), indicating similar locomotion performance in WT and mutant mice. We also carried out novel object recognition (NOR) tests to evaluate their object recognition and memory. We found that the RyR2 R4496C^+/−^ mutant mice spent significantly less time exploring the novel object than WT (Fig. 7i). Note that both RyR2 WT and the R4496C^+/−^ mutant mice showed no side preference in the NOR tests (Supplementary Fig. 15b), and that there was no significant difference in the exploration time between WT and R4496C^+/−^ mutant mice (Supplementary Fig. 15c). Collectively, these results indicate that the arrhythmogenic RyR2 R4496C^+/−^ mutation impairs hippocampal LTP, learning, and memory.

### The R4496C^+/−^ mutation has no significant impact on presynaptic short-term facilitation

**Fig. 8:**
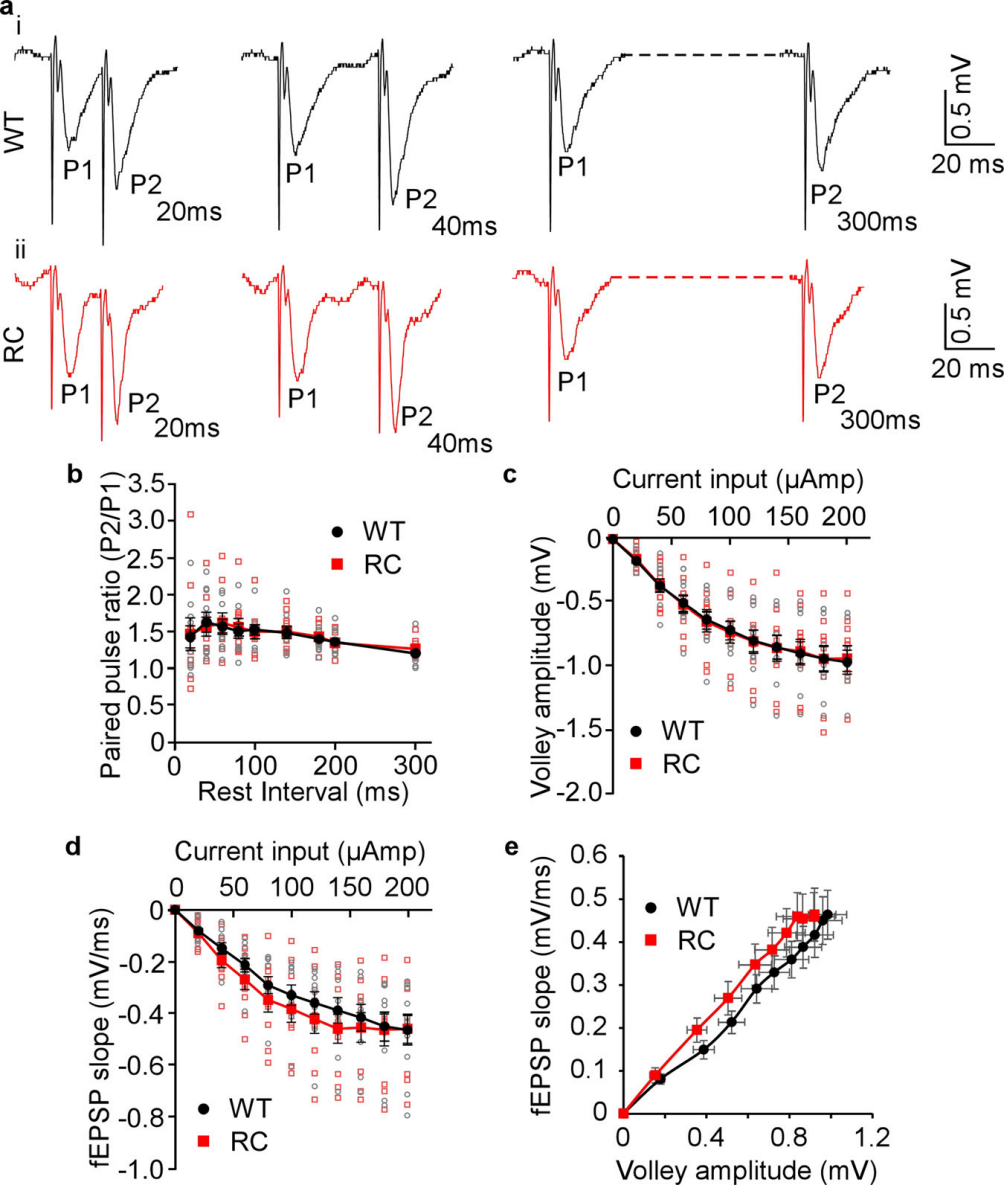
CPVT RyR2 mutation R4496C^+/−^ has no significant effect on presynaptic short-term facilitation. Representative traces of paired pulse recordings in Schaffer collaterals in dorsal hippocampal slices from RyR2 WT (**a**i) and RyR2-R4496C^+/−^ (RC) mutant (**a**ii) mice (3–4 months old). Stimulus input current for Schaffer collaterals was 60 µA. The relationship between paired pulse ratio and rest interval in WT and R4496C^+/−^ mutant brain slices is shown in **b**. Note that there are no significant differences in the amplitude of fiber volleys (**c**), or in the fEPSP slopes in relation to the current input (**d**), or fiber volley amplitude against the fEPSP slope (**e**) between the WT and RyR2 R4496C^+/−^ mutant brain slices. Data shown are mean ± SEM (*n* = 10 slices from 8 WT and 7 RC brains).

In light of the newly revealed subcellular distribution of RyR2, we also assessed the impact of the RyR2 R4496C^+/−^ mutation on presynaptic short-term plasticity of CA1 pyramidal neurons. We measured presynaptic short-term facilitation in hippocampal Schaffer collaterals in brain slices from RyR2 WT and R4496C^+/−^ mutant mice using the paired pulse stimulation protocol. As shown in Fig. 8, there was no significant difference in paired pulse facilitation between RyR2 WT and the R4496C^+/−^ mutant (Fig. 8a, b). Maximal facilitation (~160%) was observed at secondary pulses (P2) 40–60 ms after the initial pulse (P1) in both the WT and RyR2-R4496C^+/−^ mutant (Fig. 8b). There were also no significant differences in the amplitude of Schaffer fiber volleys nor in the field excitatory postsynaptic potential (fEPSP) slopes in relation to the current input between the WT and R4496C^+/−^ mutant, also no significant difference in the fiber volley amplitude against the fEPSP slope (Fig. 8c–e). Thus, the arrhythmogenic RyR2 R4496C^+/−^ mutation does not significantly affect presynaptic short-term plasticity of CA1 pyramidal neurons.

## Discussion

RyR2 is predominantly expressed in the heart and brain^60^, therefore, given this tissue distribution, defective RyR2 function is expected to cause diseases in both organs. In support, increased RyR2 function as a result of RyR2 mutations can lead to lethal cardiac arrhythmias, such as CPVT^2^, ID and cognitive deficits^11,12^. While the arrhythmogenic mechanisms of CPVT RyR2 mutations have been extensively studied, the mechanism by which these mutations cause cognitive dysfunction is largely unknown. In the present work, we determined the subcellular distribution of RyR2 in hippocampus and assessed the impact of the CPVT RyR2 R4496C^+/−^ mutation on neuronal activity, neuronal excitability, LTP, learning and memory. We found that RyR2 was predominantly detected in the soma and dendritic shafts, but not in presynaptic structures or dendritic spines of hippocampal CA1 pyramidal neurons. We also found that CPVT RyR2 R4496C^+/−^ mutation increased neuronal activity and excitability, and impaired LTP, learning and memory. These findings provide novel insights into the role of CPVT RyR2 mutations in ID.

RyRs are known to be expressed in different subcellular compartments of hippocampal neurons^6,13–17^, and are believed to play an important role in a variety of neuronal functions^14,17–19,21–26,61,62^. However, little is known about the precise subcellular distribution of the RyR2 isoform in these neurons. Although anti-RyR2 antibodies have been used to localize RyR2 in the brain^13,16^, the presence of three different RyR isoforms and the unclear specificity of these antibodies make the precise subcellular localization of RyR2 challenging. To avoid these potential problems, we developed a novel platform to localize RyR2 in brain tissue by creating a KI mouse model that expresses a GFP-tagged RyR2^31^. To amplify the GFP signal and to avoid green autofluorescence, we generated a novel AF647-conjugated, GFP-specific probe^32,33^. Using this novel platform, we determined the subcellular distribution of GFP-RyR2 by performing co-localization analysis with known neuronal subcellular markers in fixed brain slices. We also directly visualized GFP-RyR2 in tdTomato-filled hippocampal neurons in live brain slices. We found that GFP-RyR2 was predominantly localized to the soma and dendrites, but not the presynaptic terminals or dendritic spines of CA1 pyramidal neurons.

This subcellular distribution of RyR2 has important implications for the role of RyR2 in neuronal function. Although previous studies have indicated the involvement of RyRs in presynaptic transmission in hippocampal Schaffer collaterals^19,21,22,63–65^, the specific role of RyR2 in this process is unclear. Given that RyR2 is barely detected in presynaptic terminals in the stratum radiatum of the CA1 region (i.e., axonal projections from the CA3 pyramidal neurons), it would argue against a direct role of RyR2 in presynaptic short-term plasticity of CA1 pyramidal neurons. In line with this, we found that the RyR2-R4496C^+/−^ mutation did not significantly alter presynaptic short-term facilitation of CA1 pyramidal neurons. RyR2 is likewise barely detected in the dendritic spines of CA1 pyramidal neurons, which would also argue against a direct role of RyR2 in Ca^2+^ handling within dendritic spines. Therefore, RyR-related Ca^2+^ signaling previously reported in presynaptic terminals or dendritic spines of CA1 pyramidal neurons is likely attributable to other RyR isoforms (RyR1 and/or RyR3)^18–20,22,61,63,64^. It is important to note that the role of RyR2 in presynaptic short-term facilitation of CA1 pyramidal neurons may depend on developmental stages^37,66^, sample preparations (acute brain slices versus slice cultures)^18–20^, or specific brain regions^61^. In this regard, it is of interest to know that, although GFP-RyR2 clusters were hardly detected in the axonal projections from CA3 region in the distal stratum radiatum area of the CA1 region, they were detected in the large mossy fibers projecting from the DG region to the stratum lucidum area of the CA3 region. The detection of GFP-RyR2 in the CA3 mossy fibers is consistent with that reported previously^16^.

Altered RyR2 function can lead to impaired LTP and cognitive deficits^67,68^. However, the impact of CPVT RyR2 mutations on hippocampal LTP, learning, and memory is largely unknown. To this end, we employed a KI mouse model harboring the CPVT RyR2 R4496C^+/−^ mutation. We found that this mutation exerted no significant effect on presynaptic short-term facilitation of CA1 pyramidal neurons, consistent with its absence in these structures, but significantly impaired hippocampal LTP, learning, and memory, which are consistent with the ID observed in CPVT patients with RyR2 mutations. Furthermore, we found that the RyR2 R4496C^+/−^ mutation markedly decreased the A-type K^+^ current and increased the frequency of spontaneous Ca^2+^ transients and spontaneous action potential firings. These effects of the RyR2 R4496C^+/−^ mutation would lead to chronically increased neuronal excitability and persistent neuronal hyperactivity of hippocampal CA1 neurons. Interestingly, persistent reduction in the A-type K^+^ current and neuronal hyperactivity have also been observed in mouse models of Alzheimer’s disease^50,69–71^. Thus, given the critical role of neuronal excitability in LTP, learning, and memory^42–44^, our data suggest that CPVT RyR2 mutations may result in ID by impairing hippocampal LTP, learning, and memory as a result of persistent neuronal hyperactivity. It is of importance to note that the chronically reduced A-type K^+^ current as a result of the RyR2 R4496C^+/−^ mutation would be different from the neuronal activity-induced, acute reduction in A-type K^+^ current, which has been shown to facilitate the induction of LTP^72^. However, how chronically increasing neuronal excitability impairs LTP, learning and memory remains to be determined. In light of the restricted subcellular distribution of RyR2, it is unlikely that RyR2 is directly involved in early stages of LTP induction occurring in the presynaptic and postsynaptic CA1 regions. Thus, CPVT RyR2 mutations may instead affect synaptic activity through modulation of Ca^2+^ handling, neuronal activity, membrane excitability, and/or protein synthesis and trafficking in the soma and dendritic shafts.

It has recently been shown that about 8% of human patients with different CPVT RyR2 mutations also exhibited ID^12^. The mechanism underlying the low penetrance of RyR2 mutation-associated ID is unknown. A possible explanation is that different CPVT RyR2 mutations may affect the RyR2 channel and thus neuronal function to different extents, in turn resulting in variable degrees of cognitive impairment. Consistent with this idea, in vitro functional characterization revealed that ID-associated RyR2 CPVT mutants had a markedly enhanced response to activation by caffeine compared with those RyR2 mutations associated with CPVT without ID. Hence, whether a RyR2 mutation is associated with both CPVT and ID, or with CPVT without ID, may depend on the severity of the impact of the mutation on channel function. Further genetic, clinical, and basic studies would be required to better understand the link between CPVT and ID.

Study limitation: we cannot rule out the presence of minute levels of GFP-RyR2 in dendritic spines or presynaptic structures, that are below the detection limit of our imaging system. In addition, we also cannot rule out the possibility that the insertion of GFP into RyR2 may have some subtle effects on the subcellular localization of RyR2 in neurons. The GFP was inserted into RyR2 after residue Thr-1366 in the divergent region 2 of RyR2. To further minimize potential steric hindrance, the GFP sequence was flanked by 10-residue Gly-rich linkers on both sides of the GFP. We have been studying these GFP-RyR2 homozygous mice for a number of years and have not noticed any major deficiencies or abnormalities. Thus, it is unlikely that the GFP insertion grossly alters the subcellular localization of RyR2. To assess the impact of the RyR2-R4496C^+/−^ mutation on spontaneous neuronal activity, we monitored spontaneous Ca^2+^ transients in GCaMP6f-expressing CA1 neurons in acute brain slices using two-photon imaging. Although GCaMP6f, a fast, ultrasensitive Ca^2+^ sensing protein that is capable of detecting individual action potentials in neurons with high reliability, has been widely used to assess the spontaneous neuronal activity of cell populations, it may alter neuronal activity by virtue of its Ca^2+^ buffering capacity. It should be also noted that RyR2-R4496C^+/−^ is not one of the CPVT RyR2 mutations that have been clinically linked to ID. Hence, whether CPVT RyR2 mutations that are clinically associated with ID in humans can also alter neuronal excitability, learning and memory has yet to be determined. Given that both the RyR2-R4496C^+/−^ mutation and the CPVT RyR2 mutations that are clinically linked to ID are gain-of-function, it is likely that they would exert similar impact on neuronal excitability, but with different degrees of severity.

In summary, in the present study, we established a novel platform for defining the subcellular distribution of RyR2 in hippocampus. We demonstrated that RyR2 is predominantly localized to the soma and dendritic shafts, but not the presynaptic terminals or dendritic spines of hippocampal CA1 pyramidal or DG granular neurons. We also showed that a CPVT-causing RyR2 R4496C^+/−^ mutation increased neuronal excitability and impaired LTP, learning, and memory, providing new insights into CPVT RyR2 mutation-associated ID.

## Methods

### Animal studies

All animal studies were approved by the Institutional Animal Care and Use Committees at the University of Calgary and were performed in accordance with US National Institutes of Health guidelines. Adult KI mice expressing a GFP-tagged RyR2 (GFP-RyR2)^31^, heterozygous RyR2-R4496C (RC)^30^ and WT control littermates (3–4 months of age) of both sexes were used.

### Generation and labeling of GFP-specific probe

The cDNA encoding the GFP-binding protein cAbGFP4^32,33^ was amplified by PCR using a forward primer: GCACAGGTTCAACTGGTGGAAAGC and a reverse primer: TTATTATTTAGAGCTCACCGTCACCTG. The PCR fragment was then subcloned into the pET28HMT expression vector using the SspI restriction enzyme. The sequence of the PCR fragment was confirmed by DNA sequencing. The GFP-binding protein fused to the C-terminus of the polyhistidine-tagged maltose-binding-protein was overexpressed in DH5a E. coli cells and purified using the HisPur™ Ni-NTA Resin (Thermo Fisher; 8821). The recombinant fusion protein (the GFP-probe) was diluted in PBS (NaCl, 137 mM; KCl, 2.7; Na_2_HPO_4_, 10 mM; and KH_2_PO_4_, 1.8 mM; pH 7.4) and conjugated with the succinimidyl—ester of AF647 (Thermo Fisher; A20106) for at least 1 h at room temperature. Excess dye was removed using the Zeba™ Spin Desalting Columns (Thermo Fisher; 89882) and the fluorescently labeled GFP-probe (30 µg/ml) was stored at 4 degree until use.

### Preparation of brain slices for confocal and structured illumination microscopy (SIM) imaging

GFP-RyR2 mice and WT littermates (3–4 month old) were anesthetized and transcardially perfused with pre-chilled carbogen-bubbled artificial cerebrospinal fluid (aCSF) (NaCl, 125 mM; KCl, 3.25; MgCl_2_, 1.5 mM; and CaCl_2_, 1.5 mM; D-glucose, 25 mM; and NaHCO_3_, 25 mM; pH 7.4 adjusted with NaOH) or with the slicing solution (N-methyl D-glucamine 119.9 mM, KCl 2.5 mM, NaHCO_3_ 25 mM, CaCl_2_ 1.0 mM, MgCl_2_ 6.9 mM, NaH_2_PO_4_ 1.4 mM, glucose 20 mM, pH 7.4). They were then sacrificed by decapitation and whole brains were submerged rapidly into ice-cold aCSF or slicing solution, bubbled with 95% O_2_ and 5% CO_2_. For confocal and SIM, coronal sections (150 µm) were collected and fixed with paraformaldehyde (4%) in aCSF for ≤5 min at room temperature (RT). The reaction was stopped using aCSF containing glycine (0.5 M). Free floating sections were then blocked with a mixture (v/v = 50%) of BlockAid™ Blocking Solution (Molecular Probes; B10710) and Image-iT™ FX Signal Enhancer (Molecular Probes; I36933) for 1–2 h at RT. Blocked brain slices were labeled with the GFP-probe (30 µg/ml) for 30–45 min at RT, washed, mounted and imaged. For co-localization studies, the GFP-probe stained slices were permeabilized for 15 min with Triton X-100 (0.1%) and stained with Rhodamine Phalloidin (Thermo Fisher; R415) for 1 h at RT or overnight at 4 °C with primary antibodies against synaptophysin (Millipore; MAB5258—SY38 clone, 2 µg/ml), syntaxin (Enzo Life Sciences; ADI-VAM-SV013—SP6 clone, 1:50) or microtubule-associated protein 2 (MAP2)(Sigma; M1406 AP-20 clone, 1:100). Brain slices that were treated with primary antibodies were further incubated for 1–2 h at RT with rhodamine-conjugated secondary antibody (rhodamine (TRITC) AffiniPure™ goat anti-mouse IgG (H + L)) (JacksonImmuno Research Laboratories Inc.; 115-025-003, 1:200) before they were embedded in Prolong™ Gold Antifade Mountant (Thermo Fisher; P36930) for subsequent imaging.

### Confocal and structured illumination microscopy (SIM) imaging

Confocal xy imaging was performed with an inverted Nikon A1Rplus microscope system equipped with a Plan Fluor DIX H N2 ×40/1.3-NA oil immersion objective or Plan-Apochromat alpha ×60/1.27-NA oil immersion objective and selective excitation and emission filters. Large image acquisition was performed through automated fluorescence-based image stitching (50% image overlay). The fluorescence signals of GFP-RyR2 (Ex/Em 488/510 nm), rhodamine-conjugated phalloidin, anti-mouse (Ex/Em 561/594 nm), and the GFP-probe (Ex/Em 638/665 nm) were acquired at the indicated excitation and emission wave lengths. SIM imaging was performed on an inverted Carl Zeiss ELYRA LSM 780 microscope PS1 system equipped with an Plan-Apochromat DIC M27 ×63/1.4 oil immersion objective and excitation and emission filters. For each color, raw image *z*-stacks were acquired in 0.1 µm *z*-increments from a region of interest with a vertical/axial thickness of 5 µm using five rotations, five phases. Zeiss ZEN 2012 black software (Zeiss, Germany) was used for channel alignment and SIM image reconstruction.

### Preparation of brain slices for ex vivo two-photon Ca^2+^ imaging, confocal in situ imaging, and electrophysiological recordings

Acute brain slices were prepared according to the published procedures with some modifications^73^. Briefly, mice were anesthetized with isoflurane (5%) and perfused through the heart with 20 ml of ice-cold, carbogenated (95% O_2_, 5% CO_2_), N-methyl-D-glucamine (NMDG)-cutting solution, containing NMDG, 93 mM; KCl, 2.5 mM; NaH_2_PO_4_, 1.2 mM; NaHCO_3_, 30 mM; HEPES, 20 mM; glucose, 25 mM; thiourea, 2 mM; Na-ascorbate, 5 mM; Na-pyruvate, 3 mM; CaCl_2_ ∙ 2H_2_O, 0.5 mM, and MgSO_4_ ∙ 7H_2_O, 10 mM, pH to 7.3–7.4 with HCl. The brains were rapidly removed and placed in ice-cold, carbogenated cutting solution for 30 s. Transverse hippocampal slices (180–260 μm thick) were prepared using a Vibratome (Leica, VT-1200S) and incubated at 32 °C with carbogenated cutting solution. We then performed Na^+^ spike-in schedule according to the mouse age. The slices were then kept in HEPES containing aCSF (NaCl, 92 mM; KCl, 2.5 mM; NaH_2_PO_4_, 1.25 mM; NaHCO_3_, 30 mM; HEPES, 20 mM; glucose, 25 mM; thiourea, 2 mM; Na-ascorbate, 5 mM; Na-pyruvate, 3 mM; CaCl_2_ ∙ 2H_2_O, 2 mM, and MgSO_4_ ∙ 7H_2_O, 2 mM, pH to 7.3–7.4 with NaOH) at room temperature for at least 60 min before use.

### Ex vivo two-photon Ca^2+^ imaging of CA1 neurons

RyR2 WT and RyR2-R4496C^+/−^ mutant mice were cross-bred with the heterozygous Thy1-GCaMP6f transgenic mice^45,74^ (GP5.17, JAX 025393) to express the GCaMP6f Ca^2+^ sensing probe (driven by the Thy1 promotor) in hippocampal neurons. After incubating at room temperature for at least 60 min, the hippocampal slices (260 μm thick) were put under an up-right two-photon imaging system (SP8 DIVE, Leica, Germany) with CHAMELEON HEAD/PSU: ULTRA (II): 80 MHz (RoHS) laser (Coherent, UK). A ×25 water-immersion objective with NA 0.95 (Leica, Germany) was used for imaging. Laser wavelength was set at 920 nm. Images were recorded with a resolution of 296 × 296 pixels at 16.77 fps. GCaMP6f fluorescence signals from hippocampal cells in the CA1 region were continuously recorded at the resting condition (spontaneous Ca^2+^ transients) for 5 min and after the addition of KCl (50 mM) (KCl-induced Ca^2+^ transients).

### AAV9-mediated expression of tdTomato in hippocampal neurons

RyR2-GFP or RyR2 WT control (no GFP) mice were anesthetized by 1.5–2% isoflurane (Fresenius Kabi) with oxygen, and were properly mounted on a stereotaxic frame (Stoelting) with a heated pad to maintain body temperature. A dose of 2 mg/kg molexicam (Boehringer Ingelheim) was intraperitoneally injected before surgery. The animal eyes were lubricated by Optixcare Eye Lube (CLC Medica) to keep cornea moist. Hairs were shaved off, and the skin was disinfected with 75% ethanol. A minimum incision was made at the midline to expose the bregma and lambda. Adeno-associated viruses expressing tdTomato (AAV9-hSYN1-tdTomato-WPRE, titer: 2.6 × 10^12^ GC/ml) were injected at a speed of 23 nl/s using the Nanoject II Auto-Nanoliter Injector (Drummond) via a coronal window into CA1 region (anteroposterior (AP) −2.0 mm, mediolateral (ML) −1.6 mm, dorsoventral (DV) −1.5 mm; 115 nl), CA3 region (AP −1.9 mm, ML −2.5 mm, DV −2.0 mm; 46 nl), or the DG region (AP −2.0 mm, ML −1.5 mm, DV −1.8 mm; 69 nl). The injector was maintained at the site for at least 10 min after micro-injection, then removed slowly to avoid possible backflow. The incision was carefully sutured, and the mice were returned to cage once recovered from anesthesia.

### Confocal in situ imaging of hippocampal neurons of live brain slices

Acute angle hippocampal slices (180 μm) were prepared from AAV9-tdTomato injected mice 5–7 days after stereotaxic injection. The slices were incubated in 95% O_2_ and 5% CO_2_ HEPES containing aCSF solution at room temperature until use. An up-right Leica imaging system (SP8 DIVE) was used to capture the confocal x–y images (pinhole: 1 AU) with a pixel density of 4096 × 4096 (pixel size: 0.01 μm × 0.01 μm) immediately after the slices were immersed in mounting medium and sealed with a coverslip (18 mm × 18 mm × 0.17 mm). The GFP signal (excitation: 488 nm, detection: 493–545 nm) and tdTomato signal (excitation: 552 nm, detection: 559–789 nm) were acquired using the “between frames” mode.

### Electrophysiological recording of short-term plasticity and long-term potentiation in hippocampal Schaffer collaterals

Schaffer collateral fibers were stimulated in stratum radiatum at the CA3–CA2 boundary to record fEPSPs in the CA1 stratum radiatum (in ~220–280 µm distance from stratum pyramidale) of transverse dorsal slices (300 µm) from RyR2 WT and RyR2-R4496C^+/−^ mutant mice (3–4 months old). The slope of fEPSPs were determined from peaks that occurred ~2–6 ms after the stimulation to avoid potential interference from cell body population spikes as described by Low et al.^75^ and Turner et al.^76^. Paired pulse facilitation was assessed via current stimulation (60 µA) with inter-stimulus intervals of 20–300 ms. The baseline was established for at least 20 min before a series of 15 sets of two consecutive pulses was applied. fEPSP slopes of the initial pulse (P1) was set in relation to the fEPSP slope recorded in response to a secondary pulse (P2) yielding the paired pulse ratio (P2/P1) for comparison. To evaluate basal synaptic transmission, we applied different stimulation strengths (0–200 μA in steps of 20 μA) and plotted fEPSP slopes versus the current input to compare the slope of input/output curves of fEPSP. In the experiments that followed, stimulus current was adjusted so that fEPSP stabilized at 30–50% of maximum. LTP was induced using a tetanic HFS (4 trains of 100 pulses at 100 Hz, with 20-s intervals). Synaptic responses were recorded for at least 30 min after tetanization and quantified as the slope of the evoked fEPSP as percentage of the baseline. The baseline was recorded for at least 20 min. Basal excitatory synaptic transmission was assessed via comparison of input-output relations: current input versus (1) slopes of fEPSP and (2) Schaffer fiber volley amplitude.

### Single cell patching-clamp recording

Action potentials were measured in pyramidal CA1 neurons of transverse dorsal hippocampal slices (260 µm) from RyR2 WT and RyR2-R4496C^+/−^ mutant mice (3–4 months old) using whole-cell patch-clamp with an Axopatch 700B amplifier (Axon Instruments). AP firing was recorded utilizing external solution (NaCl 124 mM, KCl 2.5 mM, HEPES 5 mM, glucose 12.5 mM, MgCl_2_ 2 mM, CaCl_2_ 2 mM, pH, 7.4) and soft-glass recording pipettes (Sutter Instruments; Novato CA) filled with an internal solution (potassium gluconate 135 mM, KCl 10 mM, HEPES 10 mM, CaCl_2_ 1 mM, MgCl_2_ 1 mM, EGTA 10 mM, ATP 1 mM, GTP 0.1 mM, and pH 7.4 adjusted with KOH). The pipette resistance was 4–6 MΩ after filling with internal solution. Spontaneous AP firing by pyramidal cells in the CA1 region was recorded at RT in whole-cell current clamp mode. For the measurement of current triggered APs, APs were initiated by injecting current from 0 to 300 pA for 1 s in 10 pA steps at 10 s intervals. AP frequency was analyzed with an injecting current of 150 pA. Resting membrane potential was measured as the voltage with no injected current. Input resistance was calculated as the slope of the linear fit of the voltage–current plot between −60 and + 20 pA. Data were acquired and analyzed using pCLAMP 10.4 (Molecular Devices; Sunnyvale CA).

### A-type K^+^ current recording

Briefly, whole-cell A-type K^+^ current (*I*_A_) was elicited by depolarizing pulses to +40 mV from a holding potential of −100 mV in the presence of 20 mM tetraethylammonium and 100 nM tetrodotoxin. In steady-state activation experiments, membrane potential was held at −100 mV, and *I*_A_ was evoked by a 200-ms depolarizing pulse from a first pulse potential of −80 mV to +80 mV in 10-mV steps at 10-s intervals. Data were analyzed using the equation *G*_K_ = *I*_K_/(*V*_m_ − *V*_rev_), where *G*_K_ is the membrane K^+^ conductance, *V*_m_ is the membrane potential, and *V*_rev_ is the reversal potential for K^+^. To study steady-state inactivation of *I*_A_, currents were elicited using 1-s conditioning pre-pulses from −110 mV to 0 mV before a 200-ms test pulse of +50 mV. After normalizing each current amplitude to the maximal current, amplitude obtained from the −110 mV pre-pulse was used as a function of the conditioning pre-pulse potential and fitted with the function *I*_A_/*I*_A−max_ = 1/(1 + exp((*V*_m1/2_ − *V*_m_)/*k*)), from which, an inactivation curve of *I*_A_ was obtained, and the *V*_H_ value (the voltage at which the current amplitude was half-inactivated) was calculated. Note that in other studies and ours, the A-type K^+^ current was measured by performing whole-cell current recordings at the soma of CA1 pyramidal neurons to assess the role of somatic whole-cell A-type K^+^ current in somatic excitability.

### Afterhyperpolarization current recording

For recording the afterhyperpolarization current (*I*_AHP_), brain slices were perfused with the carbogenated aCSF and pipettes were filled with *I*_AHP_ inner solution (KMeSO_3_, 130 mM; EGTA, 0.1 mM; HEPES, 10 mM; NaCl, 7 mM; MgCl_2_, 0.3 mM; di-tris-creatine, 5 mM; Tris-ATP, 2 mM and Na-GTP, 0.5 mM, pH 7.3 with KOH). *I*_AHP_ was evoked by a 100 ms depolarizing voltage step to +60 mV from a holding potential of −85 mV. Medium (*I*_mAHP_) and slow (*I*_sAHP_) amplitudes were measured at the peak of the current and 1 s after the end of the depolarizing pulse, respectively. All cells had a resting membrane potential more hyperpolarized than −60 mV, leak current smaller than 100 pA, and an input resistance of 150–350 MΩ. Input resistance was determined from a −5 mV (100 ms) hyperpolarizing pulse applied at the beginning of each sweep. Access resistance was 80% electronically compensated and stable at <20 MΩ.

### Spatial learning and memory test

Spatial learning and memory were evaluated using the MWM task^77^. Experiments were carried out blindly. RyR2-R4496C^+/−^ mutant mice and RyR2 WT littermates were trained to localize a hidden escape platform (10 × 10 cm) in a circular pool (116.84 cm in diameter, 50 cm in depth) (San Diego Instruments, CA) via distal visual cues. The platform was submerged 1–2 cm beneath the surface in water (22–24 °C) which was rendered opaque by addition of milk powder. The localization of the pool in relation to visual cues was maintained constant during the entire task. The cues were distinct in color and size. Digital division of the tracking area (pool) into four quadrants was performed by the SMART video tracking system, Smart 3.0 (Panlab Harvard Apparatus; Barcelona Spain). The escape platform was placed in the center of the south-west quadrant for the entirety of the learning phase (4 training days) and digitally defined as target. Spatial training consisted of 4 days with 1 session comprising 5 trials per mouse. Mice were released with their heads facing the pool wall at one of four entry locations (north, east, south and west) in non-repetitive random order. Swimming was automated video-tracked until the subject found the escape platform and remained on it (≥ 5 s), or until a maximum of 1 min. Mice that did not locate the hidden platform within the time limit of 60 s were guided to the escape platform until they spent ≥10 s on it. In between trials (inter-trial interval ≥10 min), mice were housed in heated cages to avoid performance deficits due to exhaustion, or hypothermia. The latency and swimming speed to reach the escape platform were recorded for comparison. After the learning phase, memory retention was evaluated by one probe trial 24 h after the last training session. The escape platform was removed before mice were released from the north entry point into the pool. Their swimming was video-tracked for 60 s. The area at the location of the removed hidden platform was defined as target and the south-west quadrant the target quadrant. The percentage of time mice spent in the target quadrant (including the target) were measured for comparison. The mean swim speed was also determined to evaluate potential differences caused by performance dysfunction due to physical disabilities.

### Novel object recognition behavior test

RyR2-R4496C^+/−^ mutant mice and RyR2 WT littermates were habituated for 10 min per mouse in an equally illuminated, odor-free, white, plastic box (40 × 40 × 50 cm^3^) embedded with fresh aspen shavings and shreds. In between each mouse trial the box was wiped with ethanol to avoid odor-induced stress. In total, 24 h after habituation, two identical objects ware placed at equal distance to each other and the corners into the box. Each mouse was placed into the center, and allowed to move freely for 10 min. Mice were video recorded during this familiarizing phase. Side preferences was evaluated by dividing the time a mouse spent exploring one object by the time they spent at the other object. Twenty-four hours later, one of the objects was replaced by a novel object. The other object remained constant. The selection of a familiar object to be replaced was random. Each mouse was again placed into the center of the box and allowed to move freely for another 10 min while videotaped. General exploration was evaluated by determining the time exploring at the objects. The discrimination ratio describes the time a mouse explored the novel object divided by the total time it spent exploring (novel and familiar objects). The above experiments were carried out blindly.

### Computational image analysis

Basic image processing of fluorescence signals was performed using the NIS Elements AR 4.13 software (Nikon); ZEN 2012 black software (Zeiss, Germany) and Fiji (ImageJ https://imagej.nih.gov/ij/; NIH). Co-localization analysis was processed with the customized program ImageTrak (64) written by PKS. Electrophysiological recordings were acquired and analyzed using pCLAMP 10.4 (Molecular Devices; Sunnyvale CA). Behavioral video tracking was performed using SMART 3.0 (Panlab Harvard Apparatus; Barcelona Spain).

### Statistics and reproducibility

All values shown are means ± standard error of means unless indicated otherwise. For small data sets (*n* number <15) or non-Gaussian distributed data, non-parametric methods were used. For large data sets and normally distributed data, parametric tests were performed. With respect to non-parametric analyses, for experiments with two groups, Mann–Whitney *U* test was used for unpaired samples. With respect to parametric analyses, for experiments with two groups, Student’s *t* test was used for unpaired samples. Sample sizes and *p* values can be found in figure legends. *p* values smaller than 0.05 were considered statistically significant. Sample sizes for behavioral tests were estimated based on a pilot MWM test with five mice from each genotype. GPower 3.1 was used for estimating the sample sizes. For tests with two groups, two-tails Student’s *t* test was planned to be used. The effect size d value from the pilot study is 2.098, together with *α* error prob value 0.05, power value 0.95, the estimated *n* number for each group is 6.

### Reporting summary

Further information on research design is available in the Nature Research Reporting Summary linked to this article.

## Supplementary information

Supplementary Information

Description of Additional Supplementary Files

Supplementary Data 1

Reporting Summary

## Data Availability

All data used for generating the main figures can be found in Supplementary Data 1. The other data will be available upon reasonable request.

## Code availability

All code used in this study will be available upon reasonable request.
