# Supplementary material for: Subcellular localization of hippocampal ryanodine receptor 2 and its role in neuronal excitability and memory
Source: Commun Biol. 2022 Mar 1;5:183. doi: 10.1038/s42003-022-03124-2 (PMC8888588; doi:10.1038/s42003-022-03124-2)
Supplement: Supplementary file 1 — Supplementary Information [file 42003_2022_3124_MOESM1_ESM.pdf]

Supplementary Information for

Subcellular Localization of Hippocampal Ryanodine Receptor 2 and its Role in Neuronal Excitability and Memory

Florian Hiess[1,#,a], Jinjing Yao[1,$,a], Zhenpeng Song[1,%], Bo Sun[1,$], Zizhen Zhang[2], Junting Huang[2], Lina Chen[2], Adam Institoris[2], John Paul Estillore[1], Ruiwu Wang[1], Henk E.D.J. ter Keurs[3], Peter K. Stys[2,4], Grant R. Gordon[2], Gerald W. Zamponi[2], Anutosh Ganguly[5], and S. R. Wayne Chen[1,2,*]

S. R. Wayne Chen
Email: swchen@ucalgary.ca

This PDF file includes:

Figures S1 to S15

Fig. S1.

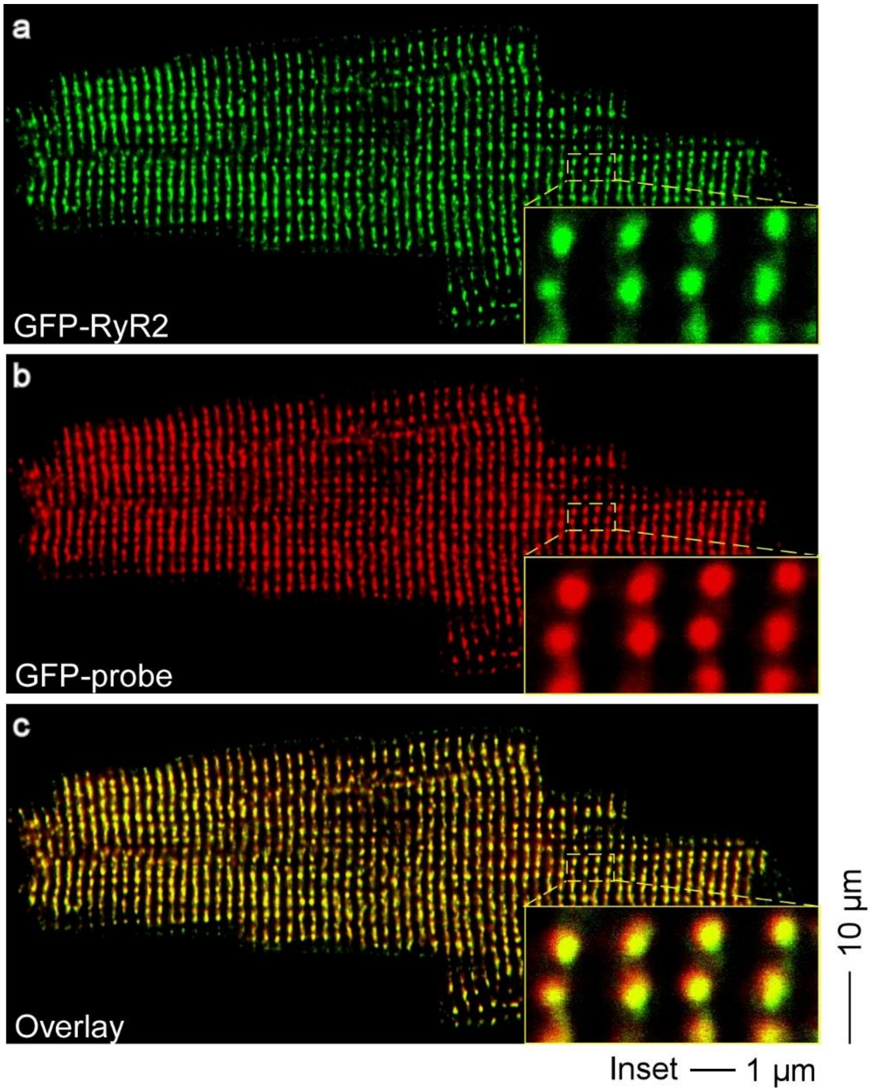

Supplemental Fig. 1 Co-localization of GFP fluorescence and GFP-probe staining in ventricular myocytes isolated from the GFP-RyR2 mice

Representative confocal images of ventricular myocytes isolated from GFP-RyR2 expressing mice. Localization of GFP-RyR2 by virtue of the GFP fluorescence (**a**) and signals of AF647 conjugated GFP-probe (**b**) showing typical single-rowed arrays of RyR2 clusters in the interior of ventricular myocytes. (**c**) Signal overlap between GFP-RyR2 and GFP-probe fluorescence.

Fig. S2.

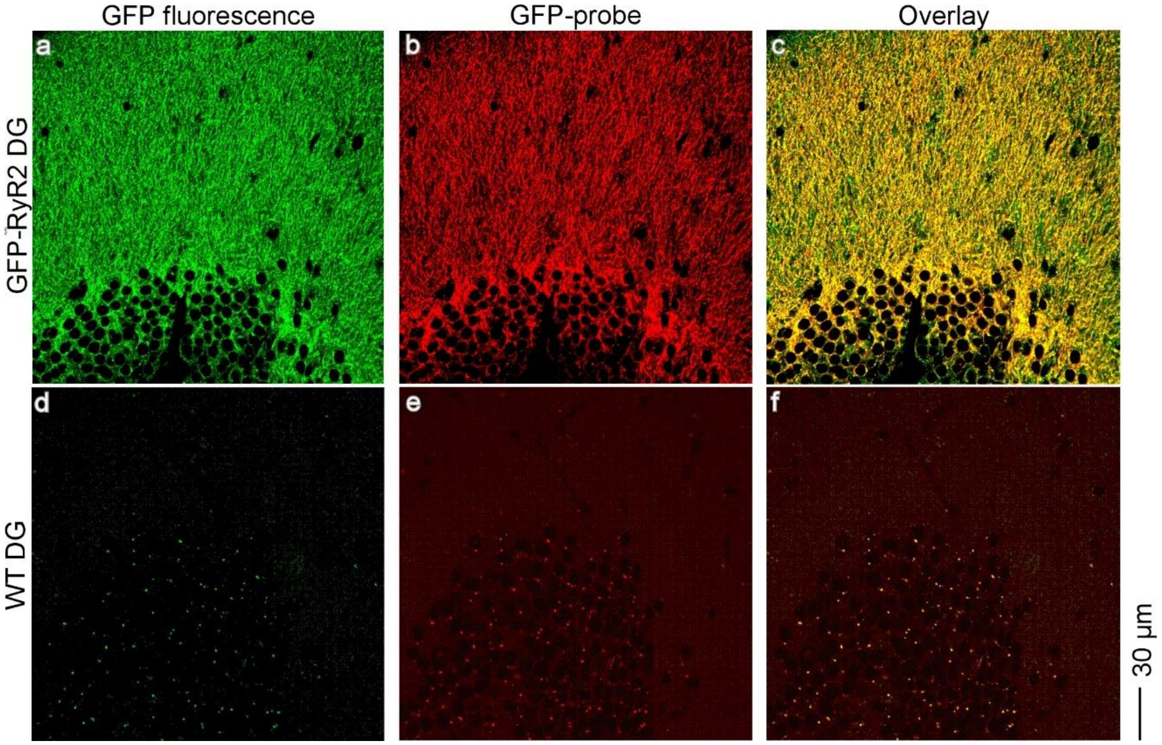

Supplemental Fig. 2 Subcellular localization of GFP-RyR2 in hippocampal dentate gyrus granular neurons in fixed brain slice

Representative confocal images of the dentate gyrus (DG) in GFP-probe labelled fixed brain slices (150 µm thick). GFP-RyR2 fluorescence (**a**) and GFP-probe signals (**b**) overlap in the dendrites and soma of granular-shaped cells in DG (**c**). There is little GFP-RyR2 fluorescence (**d**), GFP-probe signals (**e**), or overlay between these signals (**f**) in DG from WT fixed brain slices (n = 25 images from 8 brains).

Fig. S3.

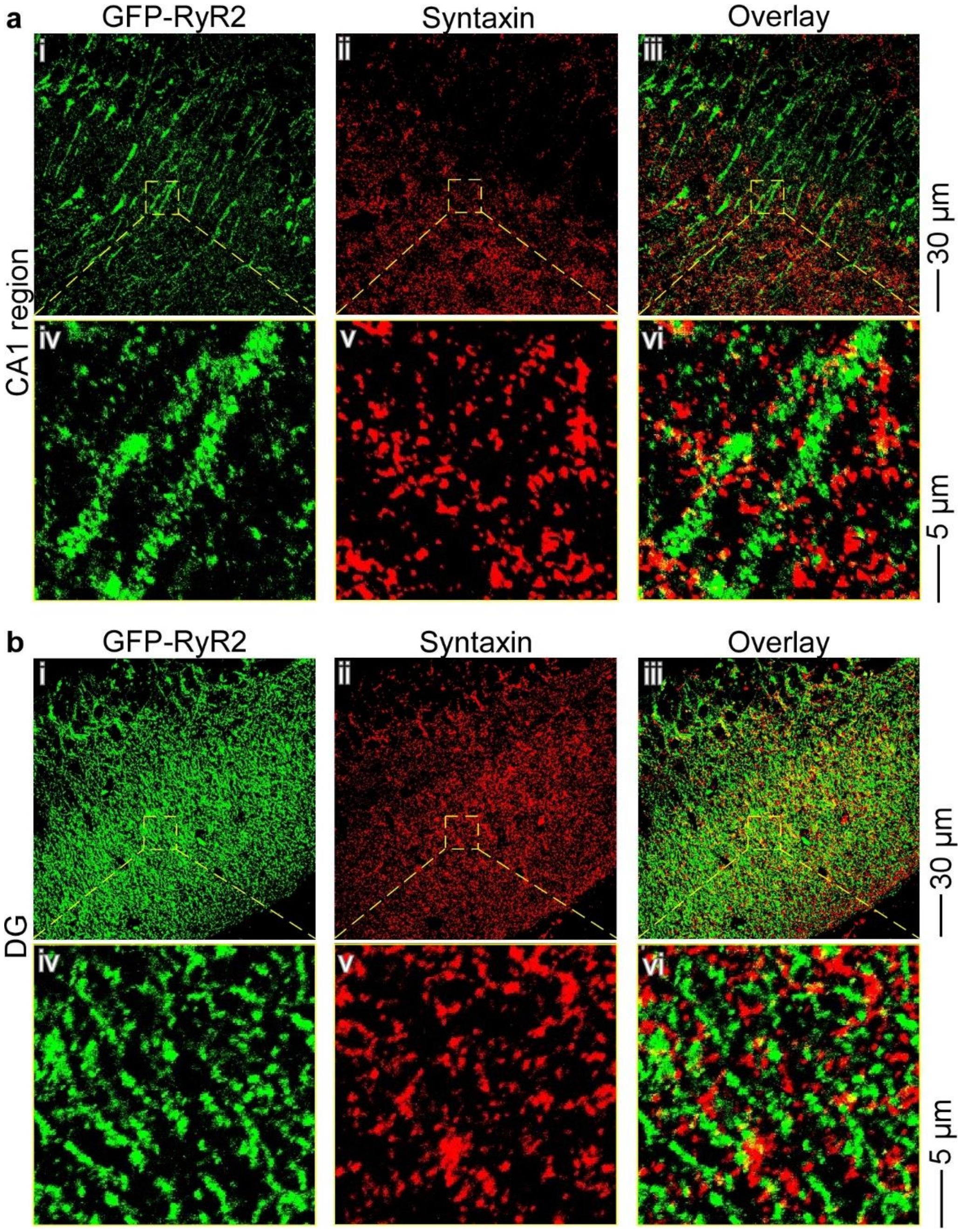

Supplemental Fig. 3 Co-localization of GFP-RyR2 with presynaptic vesicle fusion protein syntaxin in CA1 and dentate gyrus regions in fixed brain slices using confocal imaging

Representative confocal fluorescence images of hippocampal CA1 (**a**) and dentate gyrus (**b**) regions in fixed brain slices from mice expressing a GFP-tagged RyR2 that were co-

stained with the GFP-probe (displayed in green) and the presynaptic vesicle fusion protein syntaxin (n = 8 from 3 brains). GFP-probe staining (i, iv), syntaxin staining (ii, v) and overlay of the GFP-probe and syntaxin signals (iii, vi) are depicted.

Fig. S4.

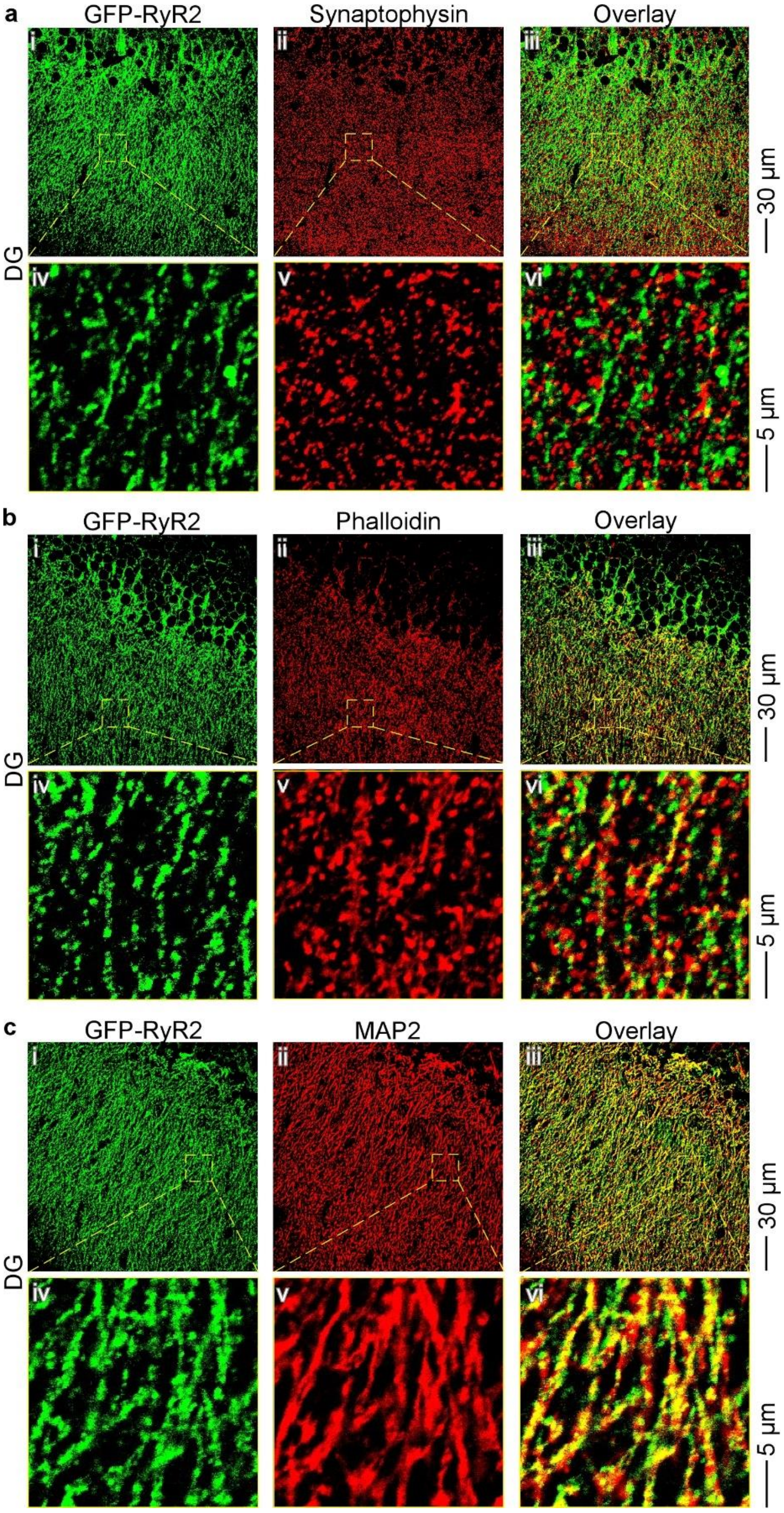
a
GFP-RyR2
Synaptophysin
Overlay
i
ii
iii
iv
v
vi
DG
30 µm
5 µm
b
GFP-RyR2
Phalloidin
Overlay
i
ii
iii
iv
v
vi
DG
30 µm
5 µm
c
GFP-RyR2
MAP2
Overlay
i
ii
iii
iv
v
vi
DG
30 µm
5 µm

Supplemental Fig. 4 Co-localization of GFP-RyR2 with synaptic markers in hippocampal dentate gyrus region in fixed brain slices using confocal imaging

Representative confocal fluorescence images of hippocampal dentate gyrus region in fixed brain slices from mice expressing a GFP-tagged RyR2 that were co-stained with the GFP-probe (displayed in green) and the presynaptic major vesicle protein synaptophysin (**a**), the F-actin targeting phalloidin (**b**), or the microtubule-associated protein 2 (MAP2) (**c**) (n = 35 imaged from 8 brains). GFP-probe staining (i, iv), synaptic marker staining (ii, v), and overlay between the GFP-probe and synaptic marker signals (iii, vi) are depicted.

Fig. S5.

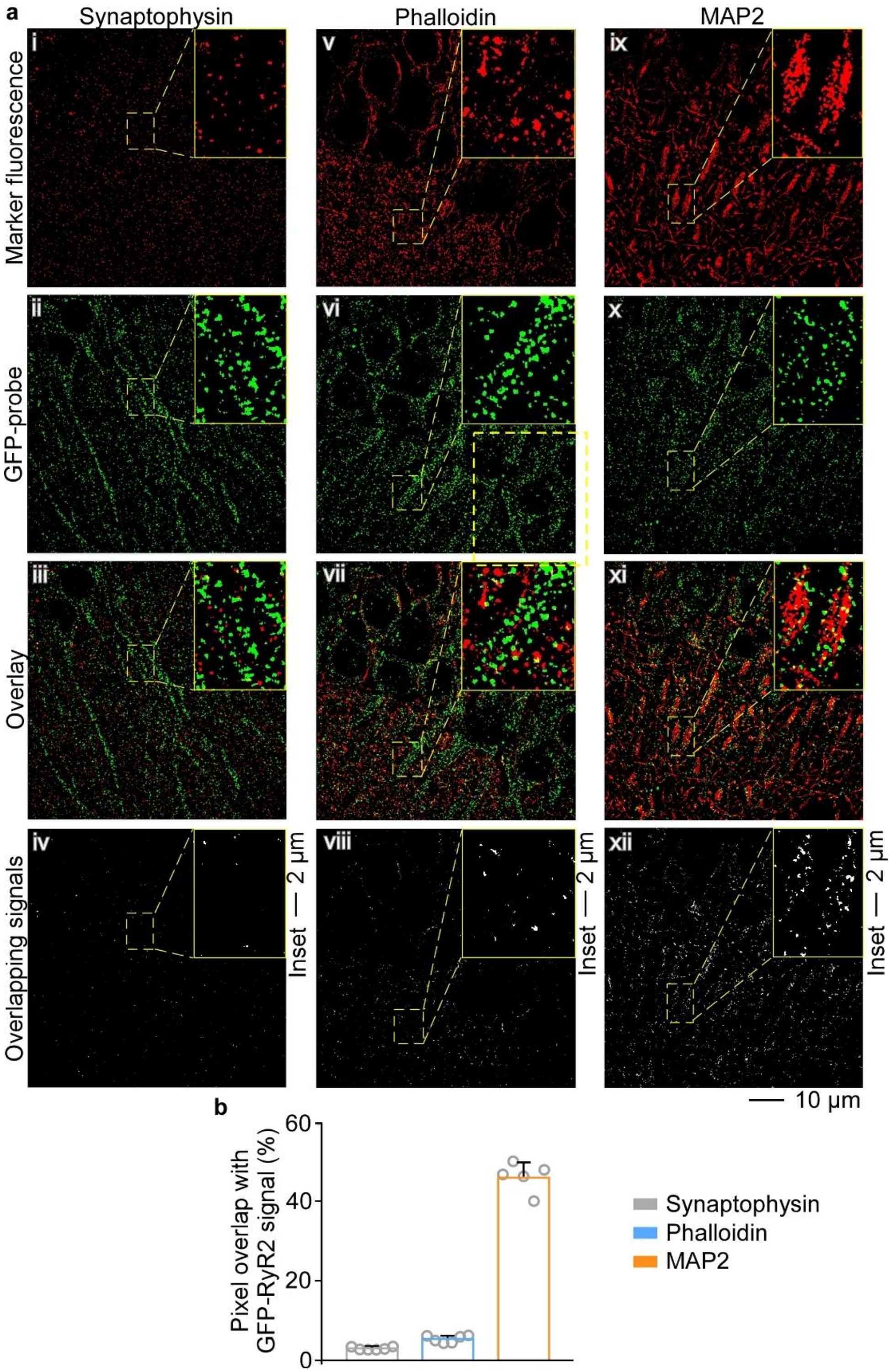

Supplemental Figure 5 Co-localization of GFP-RyR2 with synaptic markers in hippocampal CA1 region in fixed brain slices using SIM imaging

Co-localization of GFP-RyR2 and synaptic markers was determined using structured-illumination microscopic (SIM) imaging of the hippocampal CA1 region. Fixed brain slices were prepared from GFP-RyR2 mice and co-stained with the presynaptic major vesicle protein synaptophysin (**a**i-iv), the F-actin targeting phalloidin (**a**v-viii), or the microtubule-associated protein 2 (MAP2) (**a**ix-xii). The synaptic marker fluorescence (**a**i, v, ix), GFP-probe staining (**a**ii, vi, x), overlay between the GFP-probe and synaptic marker signals (**a**iii, vii, xi), and the extent of overlapping signals between the GFP-probe and synaptic markers (**a**iv, viii, xii) are shown. Overlapping signals between GFP-RyR2 and synaptophysin, phalloidin or MAP2 were quantified in panel **b**. Data shown are mean ± SEM (n = 6 images from 3 brains).

Fig. S6.

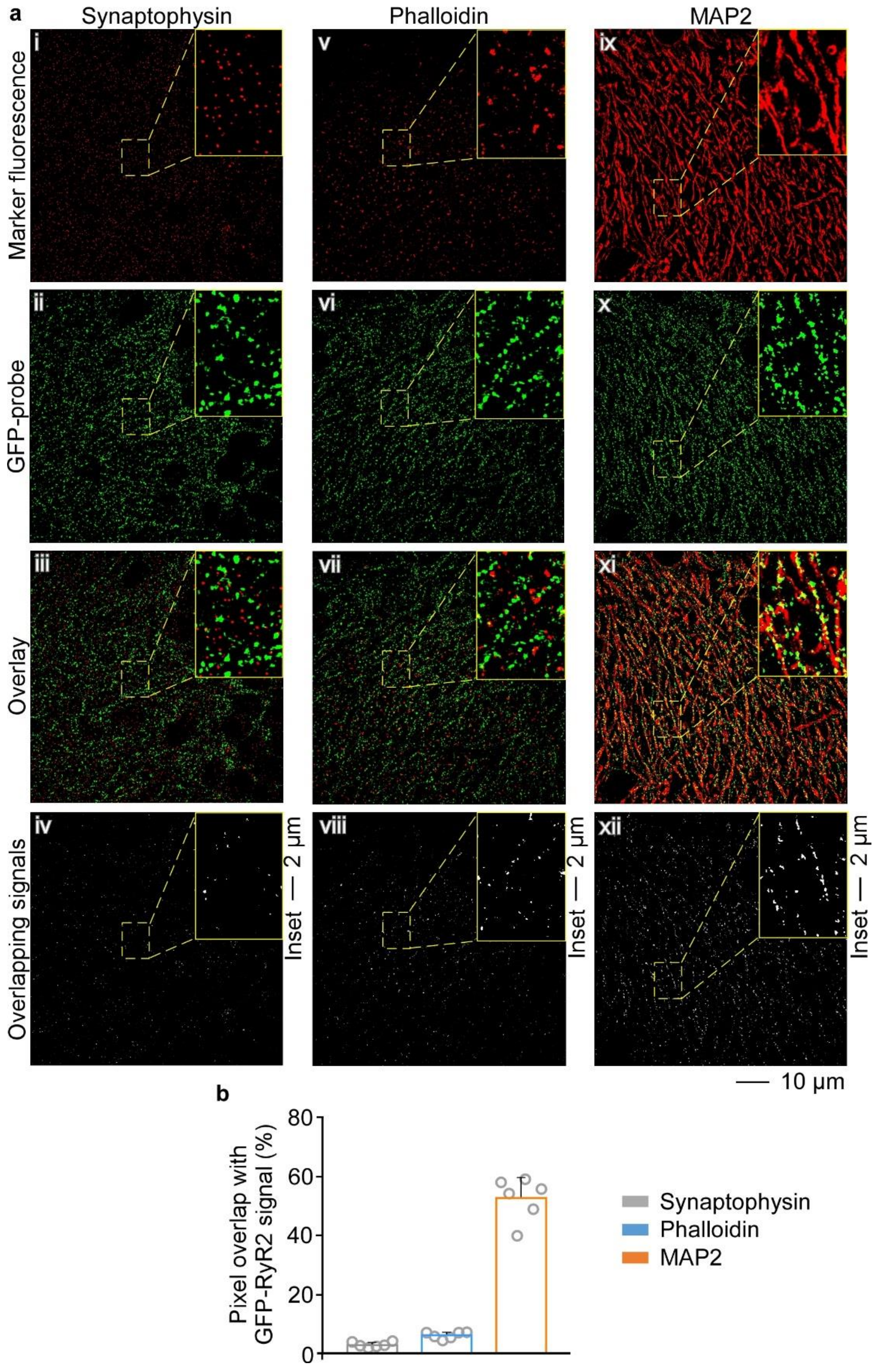

Supplemental Fig. 6 Co-localization of GFP-RyR2 with synaptic markers in hippocampal dentate gyrus region in fixed brain slices using SIM imaging

Co-localization of GFP-RyR2 and synaptic markers was determined using structured-illumination microscopic (SIM) imaging of the hippocampal dentate gyrus region. Fixed brain slices were prepared from GFP-RyR2 mice and co-stained with presynaptic major vesicle protein synaptophysin (**a**i-iv), the F-actin targeting phalloidin (**a**v-viii), or the microtubule-associated protein 2 (MAP2) (**a**ix-xii). The synaptic marker fluorescence (**a**i, v, ix), GFP-probe staining (**a**ii, vi, x), overlay between the GFP-probe and synaptic marker signals (**a**iii, vii, xi), and the extent of overlapping signals between the GFP-probe and synaptic markers (**a**iv, viii, xii) are shown. Overlapping signals between GFP-RyR2 and synaptophysin, phalloidin, or MAP2 were quantified in panel **b**. Data shown are mean ± SEM (n = 6 images from 3 brains).

Fig. S7.

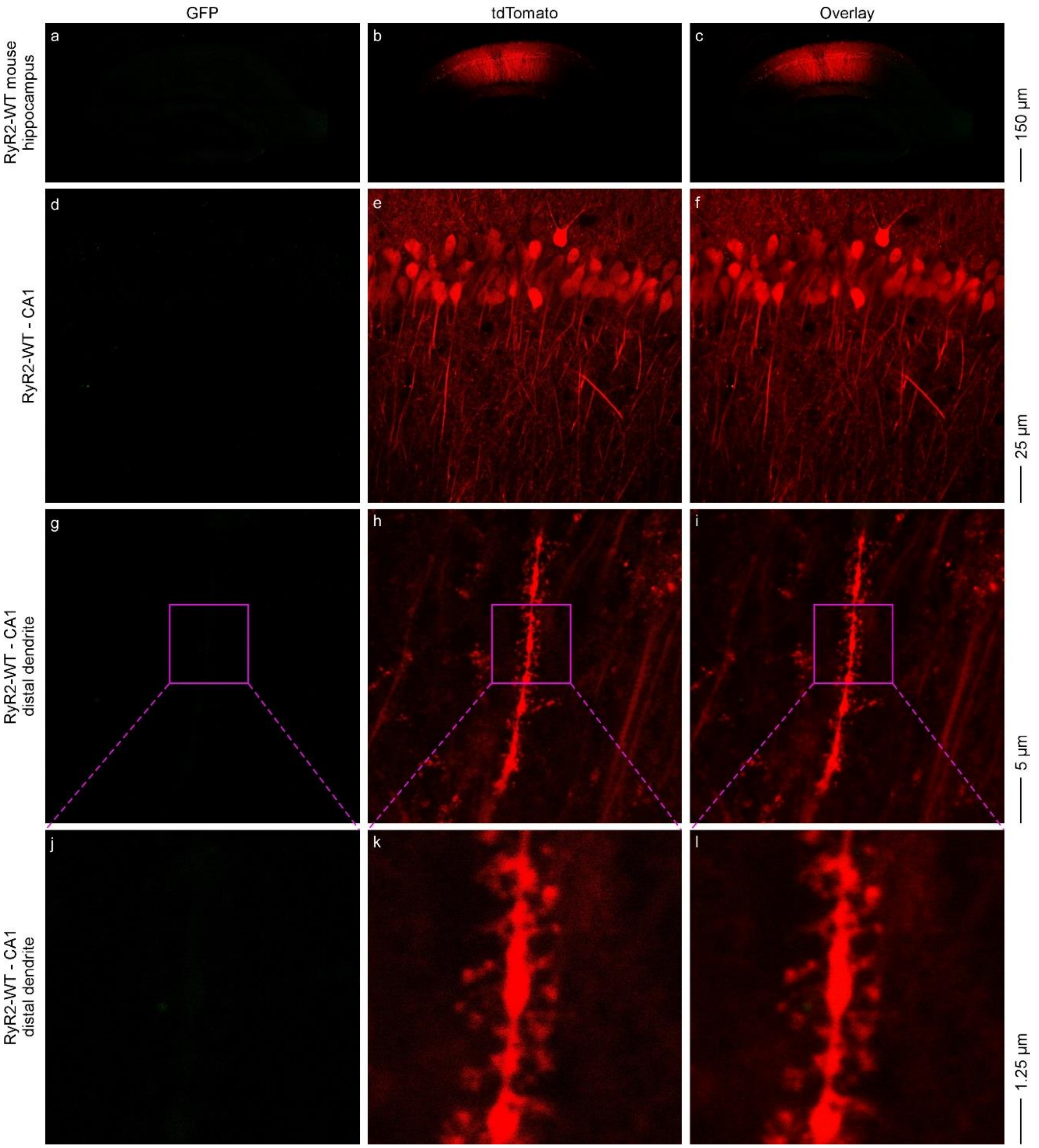

Supplemental Fig. 7 Confocal imaging of live hippocampal slices from non-GFP RyR2 WT mice with AAV9-tdTomato infected CA1 region

Representative confocal fluorescence images of live hippocampal slices from mice expressing the non-GFP RyR2 WT with AAV9-tdTomato infected CA1 region. Note that no GFP signals were detected (n = 9 images from 3 brains). Images of the whole hippocampal area showing the AAV9-tdTomato infected CA1 region (a-c). Magnified

views showing the CA1 area (d-f), a single tdTomato-filled distal dendrite (g-i), and the dendritic spines of the tdTomato-filled distal dendrite (j-l).

Fig. S8.

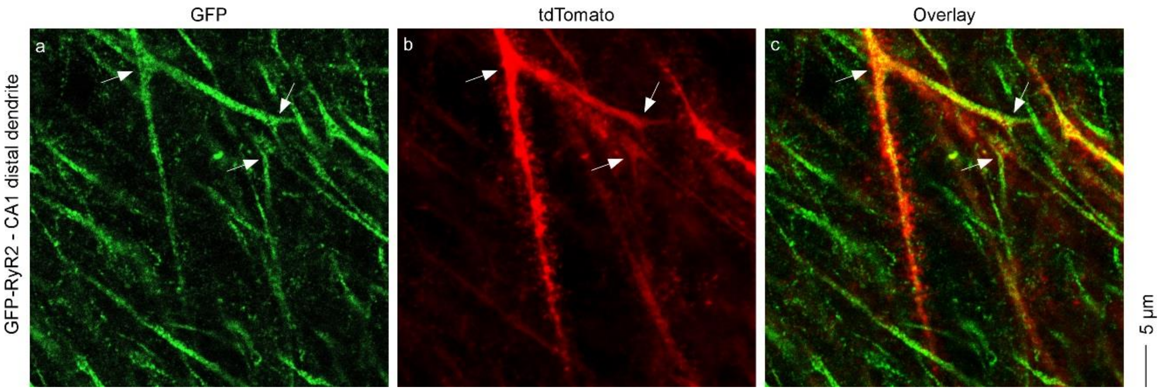

Supplemental Fig. 8 Confocal imaging of live hippocampal slices from GFP-RyR2 mice with AAV9-tdTomato infected CA1 region showing dendritic shafts and branch points

Representative confocal fluorescence images of live hippocampal slices from mice expressing the GFP-tagged RyR2 with AAV9-tdTomato infected CA1 neuron distal dendrites. (**a**) GFP-RyR2 signal from distal dendritic area of CA1 neurons. (**b**) tdTomato signal from the same area. (**c**) Signal overlap between GFP-RyR2 and tdTomato fluorescence. Arrows indicate the branch points (n = 7 images from 3 brains).

Fig. S9

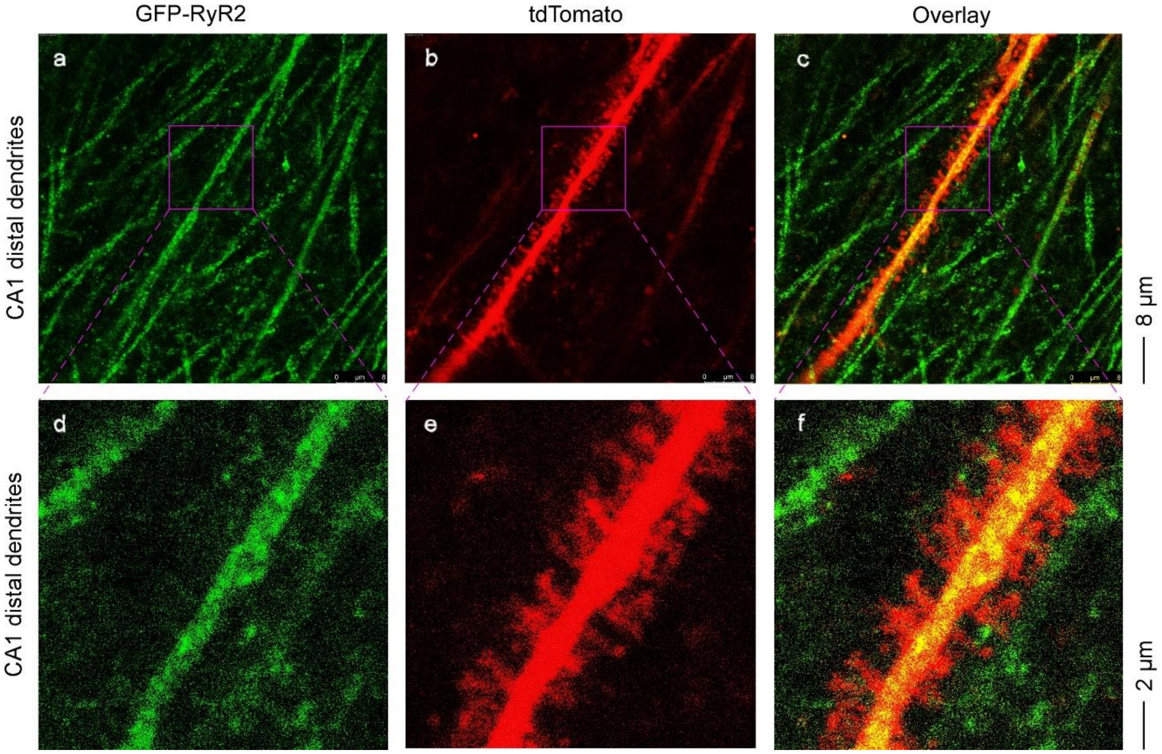

Supplemental Fig. 9 Two-photon dual imaging of live hippocampal slices from GFP-RyR2 mice with AAV9-tdTomato infected CA1 region

Representative confocal fluorescence images of live hippocampal slices from mice expressing the GFP-tagged RyR2 with AAV9-tdTomato infected CA1 region (n = 25 images from 4 brains). Magnified views showing a single tdTomato-filled distal dendrite (a-c) and the dendritic spines of the tdTomato-filled distal dendrite (d-f).

Fig. S10.

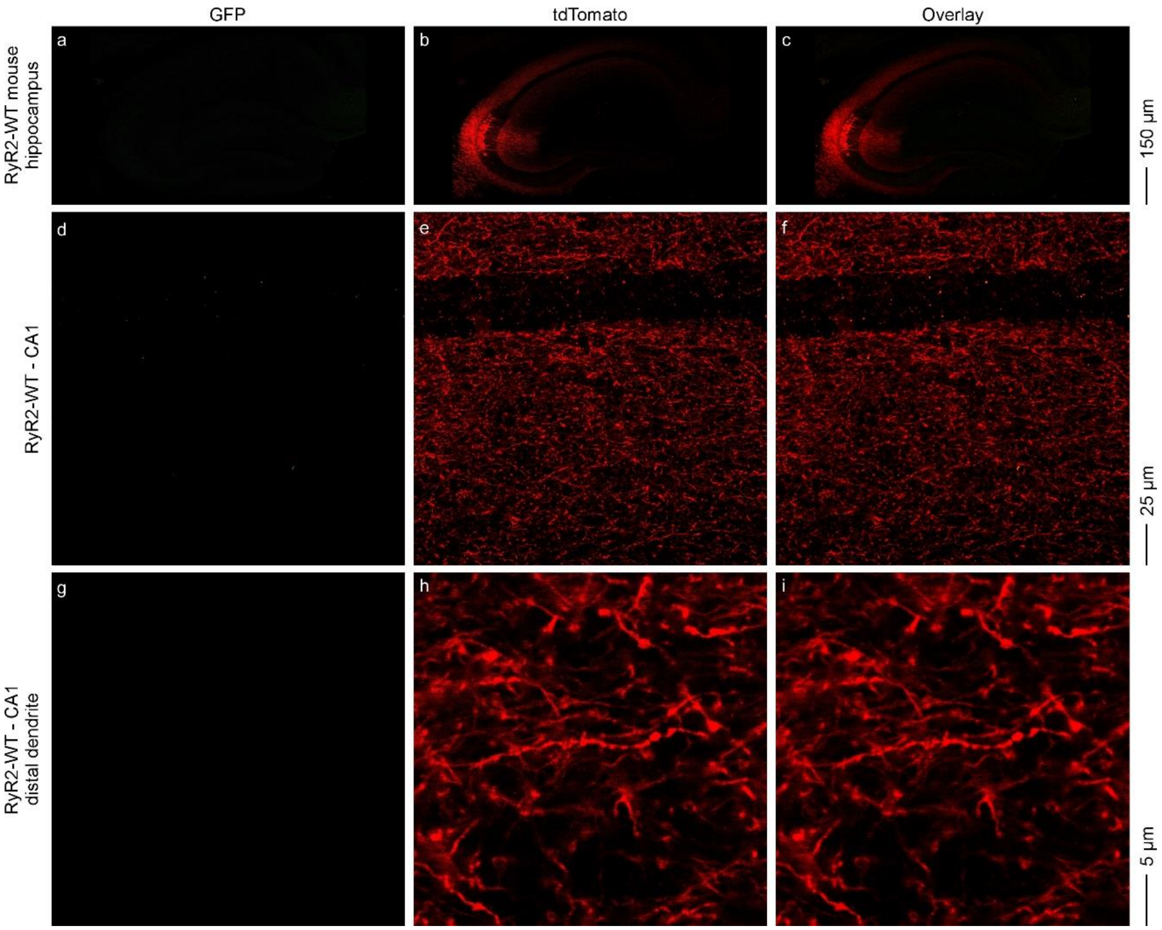

Supplemental Fig. 10 Confocal imaging of live hippocampal slices from non-GFP-RyR2 WT mice with AAV9-tdTomato infected CA3 region

Representative confocal fluorescence images of live hippocampal slices from mice expressing the non-GFP RyR2 WT with AAV9-tdTomato infected CA3 region (n = 9 images from 3 brains). Images of the whole hippocampal area showing the AAV9-tdTomato infected CA3 region (a-c). Magnified views showing the CA1 area (d-f) and the tdTomato-filled axons from CA3 region (g-i).

Fig. S11.

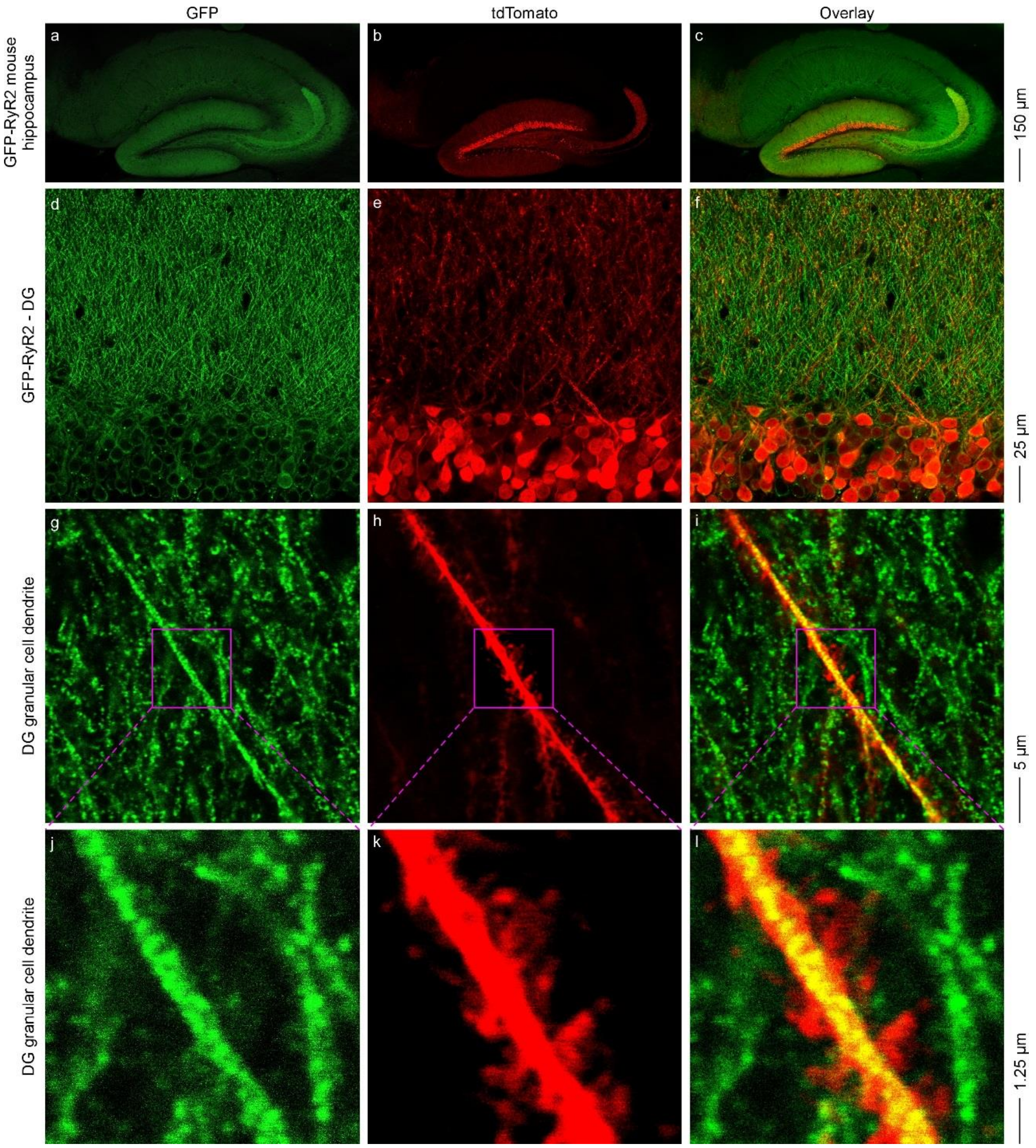

Supplemental Fig. 11 Confocal imaging of live hippocampal slices from GFP-RyR2 mice with AAV9-tdTomato infected dentate gyrus (DG) region

Representative confocal fluorescence images of live hippocampal slices from mice expressing the GFP-tagged RyR2 with AAV9-tdTomato infected DG region (n = 12 images from 4 brains). Images of the whole hippocampal area showing the AAV9-tdTomato infected DG region (a-c). Magnified views showing the DG area (d-f), a single

tdTomato-filled granular neuron dendrite (g-i), and the dendritic spines of the tdTomato-filled granular neuron dendrite (j-l).

Fig. S12.

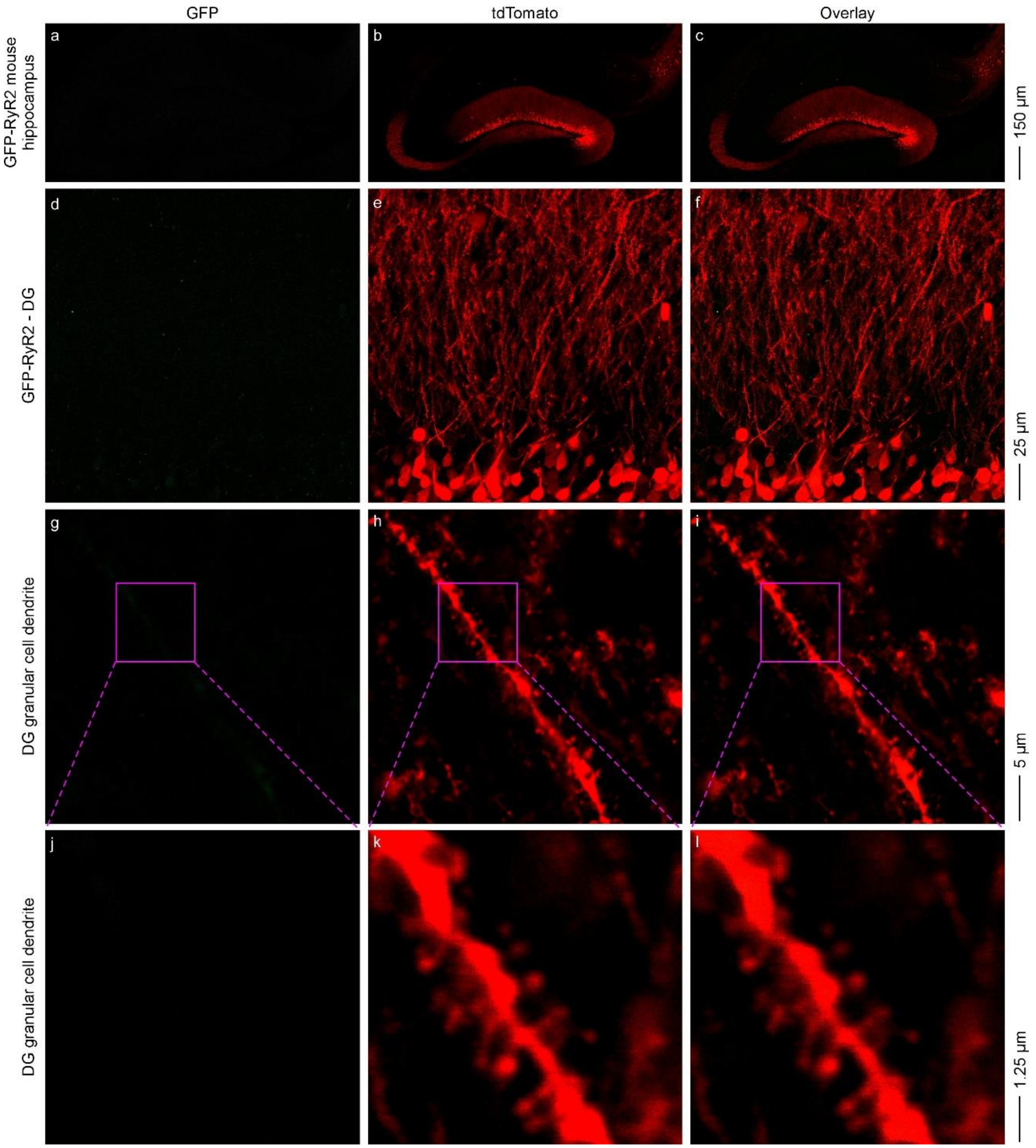

Supplemental Fig. 12 Confocal imaging of live hippocampal slices from non-GFP RyR2 WT mice with AAV9-tdTomato infected dentate gyrus (DG) region

Representative confocal fluorescence images of live hippocampal slices from mice expressing the non-GFP RyR2 WT with AAV9-tdTomato infected DG region (n = 9 images from 3 brains). Images of the whole hippocampal area showing the AAV9-tdTomato infected DG region (a-c). Magnified views showing the DG area (d-f), a single

tdTomato-filled granular neuron dendrite (g-i), and the dendritic spines of the tdTomato-filled granular neuron dendrite (j-l).

Fig. S13.

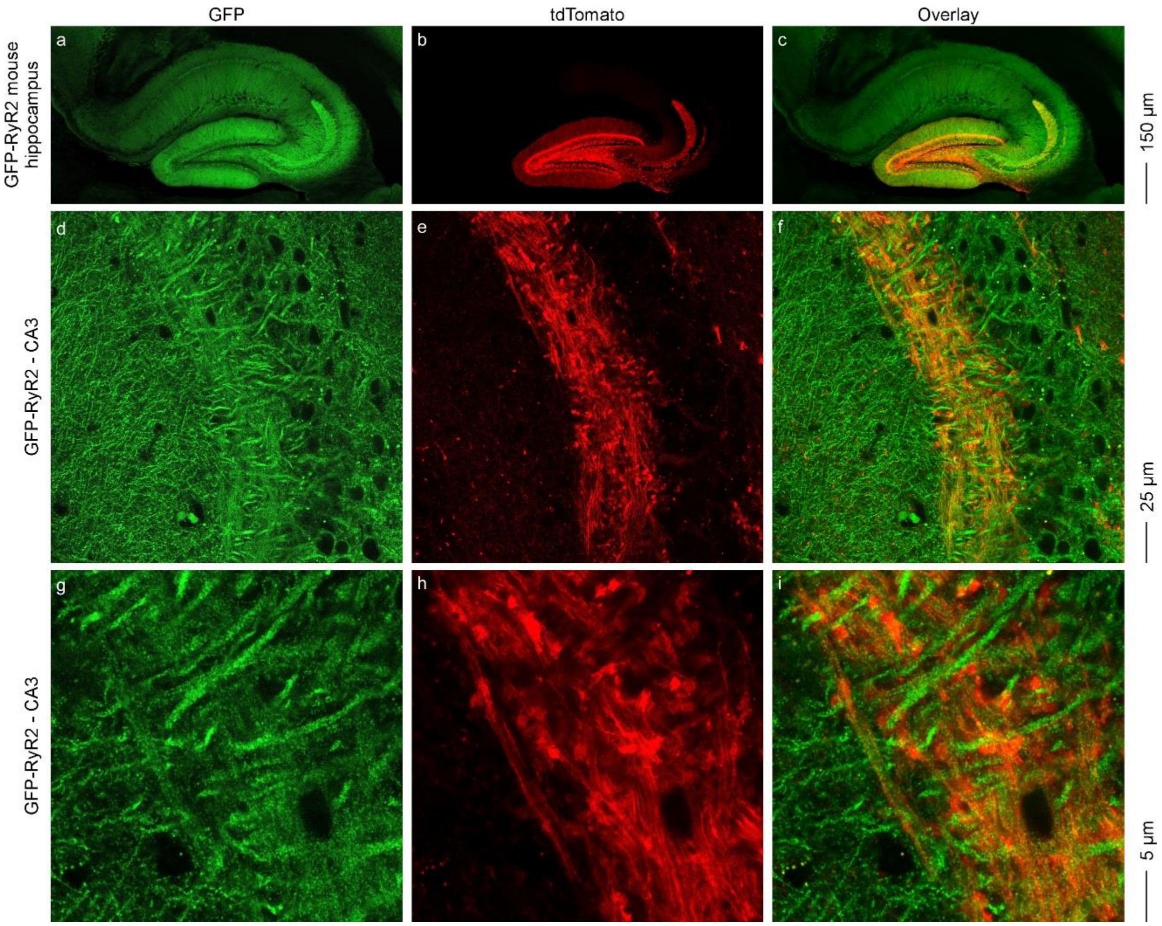

Supplemental Fig. 13 Confocal imaging of live hippocampal slices from GFP-RyR2 mice with AAV9-tdTomato infected DG granular neurons

Representative confocal fluorescence images of live hippocampal slices from mice expressing the GFP-tagged RyR2 with AAV9-tdTomato infected DG granular neurons (n = 8 images from 3 brains). Images of the whole hippocampal area showing the AAV9-tdTomato infected DG granular neurons (a-c). Magnified views showing the CA3 stratum lucidum area (d-f) and tdTomato-filled axons from the DG granular neurons and distal dendrites of CA3 neurons (g-i).

Fig. S14.

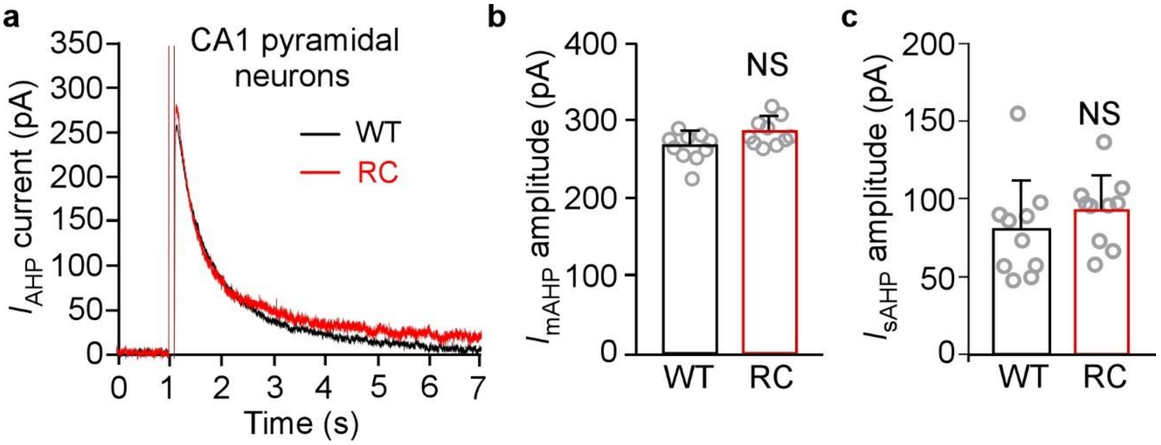

Supplemental Fig. 14 CPVT RyR2 mutation R4496C$^{+/-}$ does not affect afterhyperpolarization current (I_{AHP}) in CA1 pyramidal neurons

(**a**) Representative traces of I_{AHP} from 3-4 months old RyR2 WT and R4496C$^{+/-}$ (RC) CA1 neurons. (**b**) I_{mAHP} amplitude in 3-4 months old RyR2 WT (10 mice, 20 neurons) and R4496C$^{+/-}$ (RC) (10 mice, 20 neurons) CA1 neurons. (**c**) I_{sAHP} amplitude in 3-4 months old RyR2 WT (10 mice, 20 neurons) and R4496C$^{+/-}$ (RC) (10 mice, 20 neurons) CA1 neurons. (Mann-Whitney *U* test, NS, not significant).

Fig. S15.

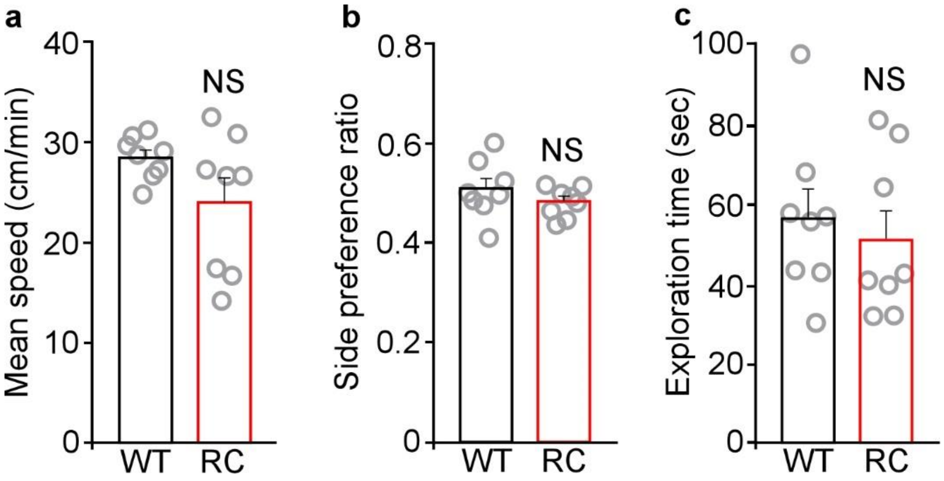

Supplemental Fig. 15 Effect of the CPVT RyR2 R4496C$^{+/-}$ mutation on swimming speed, side preference, and exploration time

The mean swimming speed (**a**) in Morris water maze test, and the side preference (**b**) and exploration time (**c**) in novel objective preference test of WT and RyR2-R4496C$^{+/-}$ mutant mice are shown. Note that there are no significant differences in these parameters between the WT and R4996C$^{+/-}$ mutant mice. Data shown are mean ± SEM (n = 8 for WT, and 8 for R4496C$^{+/-}$) (Mann-Whitney *U* test, NS, not significant).
