# Supplementary material for: Subcellular localization of hippocampal ryanodine receptor 2 and its role in neuronal excitability and memory
Source: Commun Biol. 2022 Mar 1;5:183. doi: 10.1038/s42003-022-03124-2 (PMC8888588; doi:10.1038/s42003-022-03124-2)
Supplement: Supplementary file 4 — Reporting Summary [file 42003_2022_3124_MOESM4_ESM.pdf]

nature research

Corresponding author(s): S. R. Wayne Chen
Last updated by author(s): Dec 24, 2021

Reporting Summary

Nature Research wishes to improve the reproducibility of the work that we publish. This form provides structure for consistency and transparency in reporting. For further information on Nature Research policies, see our Editorial Policies and the Editorial Policy Checklist.

Statistics

For all statistical analyses, confirm that the following items are present in the figure legend, table legend, main text, or Methods section.

n/a	Confirmed	
☐	☒	The exact sample size (*n*) for each experimental group/condition, given as a discrete number and unit of measurement
☐	☒	A statement on whether measurements were taken from distinct samples or whether the same sample was measured repeatedly
☐	☒	The statistical test(s) used AND whether they are one- or two-sided<br>*Only common tests should be described solely by name; describe more complex techniques in the Methods section.*
☐	☒	A description of all covariates tested
☐	☒	A description of any assumptions or corrections, such as tests of normality and adjustment for multiple comparisons
☐	☒	A full description of the statistical parameters including central tendency (e.g. means) or other basic estimates (e.g. regression coefficient) AND variation (e.g. standard deviation) or associated estimates of uncertainty (e.g. confidence intervals)
☐	☒	For null hypothesis testing, the test statistic (e.g. *F*, *t*, *r*) with confidence intervals, effect sizes, degrees of freedom and *P* value noted<br>*Give P values as exact values whenever suitable.*
☒	☐	For Bayesian analysis, information on the choice of priors and Markov chain Monte Carlo settings
☒	☐	For hierarchical and complex designs, identification of the appropriate level for tests and full reporting of outcomes
☒	☐	Estimates of effect sizes (e.g. Cohen's *d*, Pearson's *r*), indicating how they were calculated

Our web collection on statistics for biologists contains articles on many of the points above.

Software and code

Policy information about availability of computer code

Data collection	Images were collected by Nikon NIS Elements AR 4.13 software, Zeiss ZEN 2012 black software, Leica Application Suite X (LAS X), Electro-physiological data were collected by pCLAMP 10.4, Animal behavioral tests data were collected by Smart 3.0 (Panlab Harvard Apparatus; Barcelona Spain).
Data analysis	Colocalization analysis was processed with the customized program ImageTrak written by PKS. Calcium imaging data were analyzed with Fiji (ImageJ https://imagej.nih.gov/ij/; NIH), Electro-physiological data were analyzed by pCLAMP 10.4, Animal behavioral tests data were analyzed by Smart 3.0 (Panlab Harvard Apparatus; Barcelona Spain).

For manuscripts utilizing custom algorithms or software that are central to the research but not yet described in published literature, software must be made available to editors and reviewers. We strongly encourage code deposition in a community repository (e.g. GitHub). See the Nature Research guidelines for submitting code & software for further information.

Data

Policy information about availability of data

All manuscripts must include a data availability statement. This statement should provide the following information, where applicable:

- Accession codes, unique identifiers, or web links for publicly available datasets
- A list of figures that have associated raw data
- A description of any restrictions on data availability

All data generated in this study will be available upon reasonable request.

Field-specific reporting

Please select the one below that is the best fit for your research. If you are not sure, read the appropriate sections before making your selection.

☒ Life sciences ☐ Behavioural & social sciences ☐ Ecological, evolutionary & environmental sciences

For a reference copy of the document with all sections, see nature.com/documents/nr-reporting-summary-flat.pdf

Life sciences study design

All studies must disclose on these points even when the disclosure is negative.

Sample size	Sample sizes for behavioral tests were estimated based on a pilot MWM test with 5 mice from each genotype or treatment group. GPower 3.1 (RRID:SCR_013726) was used for estimating the sample sizes.
Data exclusions	No data were excluded.
Replication	For confocal imaging with brain slices, 3 slices from the same mouse were used for analyze. For in vitro calcium imaging with brain slices, 3 slices from the same mouse were used for analyzing. For whole-cell patch-clamp recording, at least 3 neurons from each brain were included for analyzing. For LTP recording, 1 or 2 slices from each mouse were included for analyzing.
Randomization	Mice were divided into groups with simple randomization.
Blinding	Animal allocation and experiments were performed with different person. Animal genotypes were revealed after the analyze.

Reporting for specific materials, systems and methods

We require information from authors about some types of materials, experimental systems and methods used in many studies. Here, indicate whether each material, system or method listed is relevant to your study. If you are not sure if a list item applies to your research, read the appropriate section before selecting a response.

Materials & experimental systems

n/a	Involved in the study	
☐	☒	Antibodies
☒	☐	Eukaryotic cell lines
☒	☐	Palaeontology and archaeology
☐	☒	Animals and other organisms
☒	☐	Human research participants
☒	☐	Clinical data
☒	☐	Dual use research of concern

Methods

n/a	Involved in the study	
☒	☐	ChIP-seq
☒	☐	Flow cytometry
☒	☐	MRI-based neuroimaging

Antibodies

Antibodies used	Primary antibodies against synaptophysin (Millipore; MAB5258 - SY38 clone), syntaxin (Enzo Life Sciences; ADI-VAM-SV013 – SP6 clone), and microtubule-associated protein 2 (MAP2)(Sigma; M1406 AP-20 clone). Rhodamine-conjugated secondary antibody (rhodamine (TRITC) AffiniPure™ goat anti-mouse IgG (H+L)) (JacksonImmuno Research Laboratories Inc.; 115-025-003)
Validation	For primary antibodies against synaptophysin (Millipore; MAB5258 - SY38 clone), its species reactivity is "Avian, Bovine, Fish, Human, Mouse, Rat", Key applications are: FC, ICC, IHC, WB.<br>For primary antibodies against syntaxin (Enzo Life Sciences; ADI-VAM-SV013 – SP6 clone),its species reactivity is "Human, Mouse, Rat, Bovine", applications are: IHC, WB.<br>For primary antibodies against microtubule-associated protein 2 (MAP2)(Sigma; M1406 AP-20 clone), its species reactivity is "Xenopus, bovine, human, mouse, rat, aquatic salamander, quail", applications are: IHC, WB.

Animals and other organisms

Policy information about studies involving animals; ARRIVE guidelines recommended for reporting animal research

Laboratory animals	Adult knock-in mice expressing a GFP-tagged RyR2 (GFP-RyR2), heterozygous RyR2-R4496C (RC) and wild type (WT) control littermates (3-4 months of age) of both sexes were used.
Wild animals	The study did not involve wild animals.
Field-collected samples	The study did not involve samples collected from field.

Ethics oversight	All animal studies were approved by the Institutional Animal Care and Use Committees at the University of Calgary and were performed in accordance with US National Institutes of Health guidelines.

Note that full information on the approval of the study protocol must also be provided in the manuscript.
